# TL-532, a novel specific Toll-like receptor 3 agonist rationally designed for targeting cancers: discovery process and biological characterization

**DOI:** 10.15698/mic2023.06.797

**Published:** 2023-04-19

**Authors:** Sylvain Thierry, Sarah Maadadi, Aurore Berton, Laura Dimier, Clémence Perret, Nelly Vey, Saïd Ourfali, Mathilde Saccas, Solène Caron, Mathilde Boucard-Jourdin, Marc Colombel, Bettina Werle, Marc Bonnin

**Affiliations:** 1 TOLLYS SAS, 60F avenue Rockefeller, Lyon, France; Centre Léon Bérard, Cancer Research Center of Lyon, Lyon, France.; 2 Service d'Urologie et Chirurgie de la Transplantation, Hospices Civils de Lyon, Lyon, France. Université Claude Bernard Lyon 1; TOLLYS SAS, 60F avenue Rockefeller, Lyon, France; Centre Léon Bérard, Cancer Research Center of Lyon, Lyon, France.; 3 Service d'Urologie et Chirurgie de la Transplantation, Hospices Civils de Lyon, Lyon, France; Univ Lyon, Université Claude Bernard Lyon 1.

**Keywords:** Toll-like receptor 3, agonist, cancer therapy, apoptosis, inflammation

## Abstract

Toll-like receptor 3 (TLR3) is an innate immune receptor that recognizes double-stranded RNA (dsRNA) and induces inflammation in immune and normal cells to initiate anti-microbial responses. TLR3 acts also as a death receptor only in cancer cells but not in their normal counterparts, making it an attractive target for cancer therapies. To date, all of the TLR3-activating dsRNAs used at preclinical or clinical stages have major drawbacks such as structural heterogeneity, toxicity, and lack of specificity and/or efficacy. We conducted the discovery process of a new family of TLR3 agonists that are chemically manufactured on solid-phase support and perfectly defined in terms of sequence and size. A stepwise discovery process was performed leading to the identification of TL-532, a 70 base pair dsRNA that is potent without transfection reagent and is highly specific for TLR3 without activating other innate nucleic sensors such as RIG-I/MDA5, TLR7, TLR8, and TLR9. TL-532 induces inflammation in murine RAW264.7 myeloid macrophages, in human NCI-H292 lung cancer cells, and it promotes immunogenic apoptosis in tumor cells *in vitro* and *ex vivo* without toxicity towards normal primary cells. In conclusion, we identified a novel TLR3 agonist called TL-532 that has promising anticancer properties.

## INTRODUCTION

Toll-like receptors (TLRs) are immune receptors that recognize pathogen-associated molecular patterns and are essential for generating anti-microbial immune responses. Among them, TLR3 senses endosomal double-stranded RNA (dsRNA) and is expressed in a variety of cell types including epithelial and immune cells [1, 2], and cancer cells of several histotypes [3]. Initiation of the signal transduction by TLR3 requires dimerization of its ectodomains following interaction with dsRNA in endolysosomes [4, 5].

TLR3 is an attractive target for cancer therapies [6]. It behaves as an immunomodulator of the tumor microenvironment [7], acting through cancer cells, peritumoral tissues, and immune cells by inducing Th-1 responses [8–11]. TLR3 signaling results in NF-κB, MAPK, IRF3/7 transcription factors activation leading to proinflammatory cytokines secretion, including type I interferon and chemokines [12] which recruit and activate immune cells [13]. In addition, TLR3 acts directly as a death receptor [14] in cancer cells of various origins including breast [15], melanoma [16], mesothelioma [17], head and neck [18], prostate [19], renal carcinoma [20], colon [21], oral, and lung cancer cells [3].

Numerous clinical trials have confirmed the ability of TLR3 agonists to promote antitumor activity (reviewed in [6]). In most preclinical and epidemiological studies, high TLR3 expression and/or activation is associated with a favorable prognosis, including in bladder cancer [22], mesothelioma [17], NSCLC [23], HCC [2], and breast cancer carcinoma [24].

To date, no TLR3 agonists have reached market approval in the treatment of cancer due to systemic toxicity [25], limited efficacy [6], lack of structural homogeneity [26, 27], and most importantly lack of TLR3 specificity. Indeed, molecules such as poly(I:C) and its derivatives, initially considered to be specific for TLR3, are now known to also activate cytosolic sensors, notably MDA5 and RIG-I [28, 29].

For cancer therapy, the ideal TLR3 agonist should trigger immunogenic tumor cell apoptosis and stimulate myeloid cells, thereby inducing anti-tumor immune response [6, 30]. In this study, we performed a rational design of a new family of TLR3-specific agonists that are chemically synthesized on solid-phase support, fully defined in terms of sequence and size, and active without requiring transfection agents. The discovery process addressed various parameters including length [31, 32] and composition [33]. For biological screening, the activity of the ligands was tested on both NCI-H292 human lung cancer cells and on RAW264.7 murine macrophages.

Within this new family, TL-532 strikes the best balance between inflammatory cancer cell death and cytokine induction in the myeloid cell model. In addition, TL-532 activity is strictly dependent on TLR3, induces immunogenic caspase-mediated apoptosis of cancer cells and shows no toxicity towards primary normal cells. Overall, these results indicate that TL-532 is a promising molecule for future anticancer therapeutic strategies targeting TLR3.

## RESULTS

### Rational design of TLR3 agonists

We synthesized 59 dsRNA sequences (Supplementary Tables S1 and S2) and tested their capacity to induce mTNFα secretion by RAW264.7s and apoptosis or hIL-6 secretion by NCI-H292s. Poly(I:C) high molecular weight (HMW) and poly(A:U) HMW were used as controls of TLR3 activation [28].

**Figure 1:**
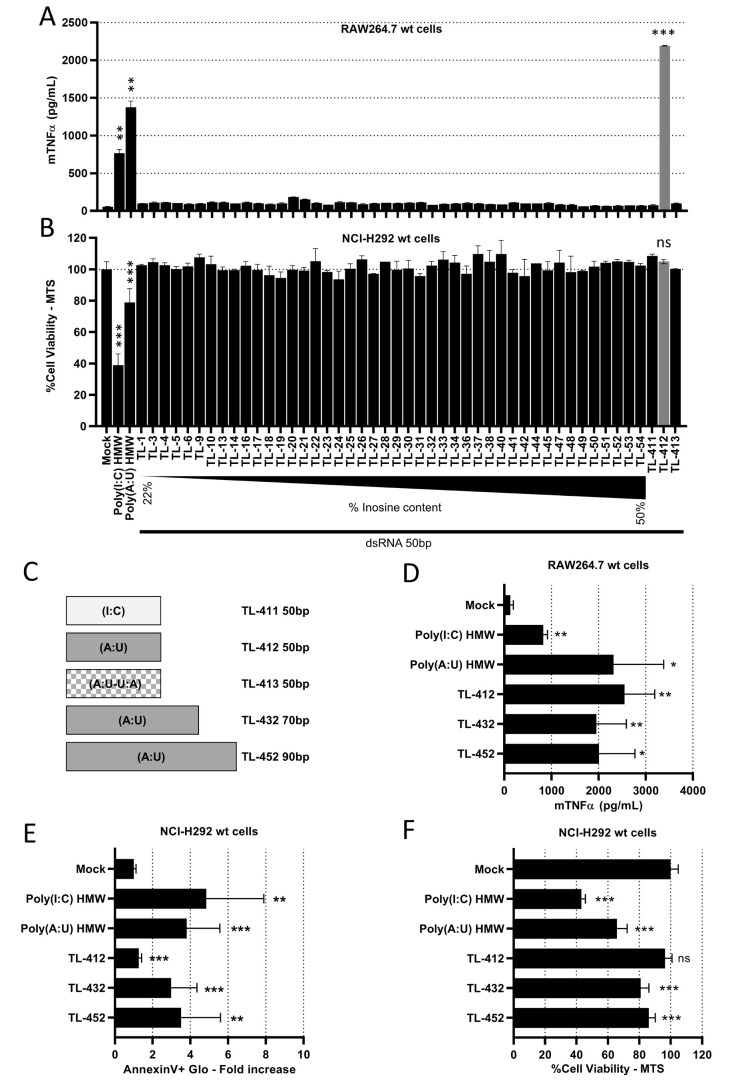
FIGURE 1: Poly(A:U) 50 bp TL-412 increases mTNFα secretion by RAW264.7 wt cells, while Poly(A:U) 70 bp TL-432 and 90 bp TL-452 trigger biological responses in both RAW264.7 wt and NCI-H292 wt cells. **(A, D)** Activation of myeloid cells by TLR3-ligands was determined on RAW264.7 cells treated with the different molecules at 10 µg/mL for 24 hours. The concentration of mTNFα was measured by ELISA. **(B, E, F)** Activation of tumor cells by TLR3-ligands was determined on NCI-H292 cells treated with the different molecules at 50 µg/mL for 24 hours. Cell viability was measured with MTS assay and expressed as percentage of untreated cells (B, F). Apoptosis was measured with AnnexinV-Glo assay and expressed as fold increase of untreated cells (E). **(A)** Among 47 tested 50 bp dsRNAs, only the Poly(A:U) (TL-412) induces mTNFα secretion by RAW264.7 wt cells. **(B)** None of the tested 50 bp dsRNAs reduces cell viability in NCI-H292 wt. **(C)** Schematic representation of a subset of dsRNA ligands, showing their sizes and compositions. TL-413, made of a random sequence containing A-U nucleotides, is represented in square. **(D)** Increasing the size of Poly(A:U) from 50 to 70 or 90 bp does not increase mTNFα secretion by RAW264.7 wt cells. **(E, F)** Increasing the size of Poly(A:U) from 50 to 70 or 90 bp triggers apoptosis and reduces cell viability in NCI-H292 wt. Data are representative of one (B) or at least two (A) independent assays or are the means of three independent assays (D-F). Results are expressed as mean ± SD. Unpaired Student's t-test: * = p<0.05; ** = p<0.01; *** = p<0.001; ns: not significant. Unpaired Student's t-test values are compared to Mock condition unless otherwise stated.

First, 44 random 50 base pairs (bp)-long dsRNA sequences (TL-1 to TL-54 in Supplementary Table S1) with increasing inosine content were generated. Three additional 50 bp dsRNAs were designed, two homopolymers Poly(I:C) (TL-411) and Poly(A:U) (TL-412), and a random Poly(A:U-U:A) sequence (TL-413). We confirmed that migration on native PAGE showed only one main band at the expected size, unlike heterogenous Poly(A:U) HMW (see Supplementary Table S1, **Fig. 1C**, and Supplementary Fig. S1A). When added at 10 µg/mL to RAW264.7s, only TL-412 Poly(A:U) induced mTNFα secretion (**Fig. 1A**), while TL-413 Poly(A:U-U:A) had no effect showing that induction of mTNFα secretion does indeed depend on nucleotide sequence. However, when added at 50 µg/mL to NCI-H292s, none of the tested sequences including TL-412 reduced cell viability (**Fig. 1B**) or induced hIL-6 secretion (Supplementary Fig. S1B).

Given the known relationship between dsRNA length and TLR3 activation [32], we next increased the length of the Poly(A:U) homopolymer from 50 bp (TL-412) to 70 bp (TL-432) and 90 bp (TL-452). We again confirmed that migration on native PAGE showed one main band at the expected size (**Fig. 1C** and Supplementary Fig. S1C). While increasing the size of poly(A:U) did not affect mTNFα secretion by RAW264.7s (**Fig. 1D**), induction of cell death, apoptosis, and hIL-6 secretion were now observed in NCI-H292s (**Figs. 1 E-F** and Supplementary Fig. S1D).

**Figure 2:**
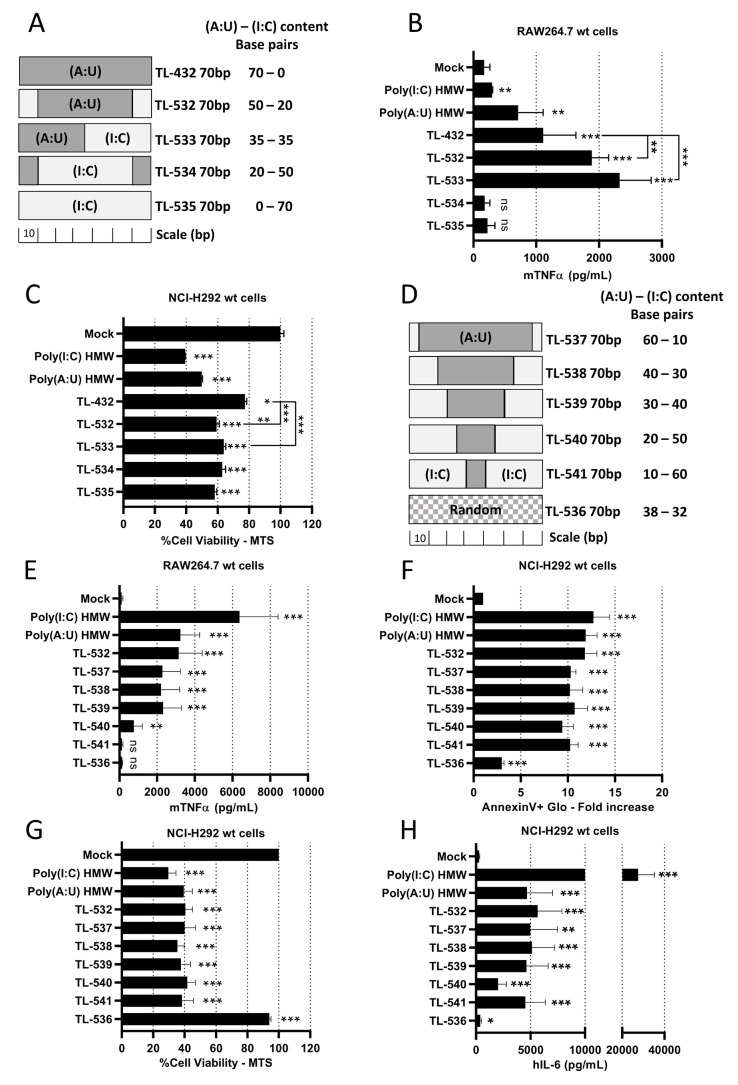
FIGURE 2: TL-532 induces the best biological balance between inflammation in RAW264.7 wt cells and tumor inflammatory cell death in NCI-H292 wt cells. **(A, D)** Schematic representation of the 70 bp dsRNAs showing their differing (A:U) and (I:C) bp content. **(B, E)** Activation of myeloid cells by TLR3-ligands was determined on RAW264.7 cells treated with the different molecules at 10 µg/mL for 24 hours. The concentration of mTNFα was measured by ELISA. **(C, F, G, H)** Activation of tumor cells by TLR3-ligands was determined on NCI-H292 cells treated with the different molecules at 500 µg/ml (C), 100 µg/ml (F, G), or 10 µg/ml (H) for 24 hours. Cell viability was measured with MTS assay and expressed as percentage of untreated cells (C, G). Apoptosis was measured with AnnexinV-Glo assay and expressed as fold increase of untreated cells (F). The concentration of hIL-6 was measured by ELISA (H). **(B, C)** Among the tested molecules, TL-532 and TL-533 induce the highest biological responses in both RAW264.7 wt and NCI-H292 wt cells. **(E)** Increasing (I:C) bp content reduces RAW264.7 wt cells activation. TL-536 is inactive in RAW264.7 wt, unlike TL-532. **(F-H)** Increasing (I:C) bp content does not impact the induction of apoptosis, viability reduction, and hIL-6 secretion in NCI-H292 wt cells except for TL-540, which induces less hIL-6 secretion comparatively. TL-536 is barely active in NCI-H292 wt cells, contrary to the TL-532. Data are the representative (C) or are the mean of three independent experiments (B, E-H). Results are expressed as mean ± SD. Unpaired Student's t-test: * = p<0.05; ** = p<0.01; *** = p<0.001; ns: not significant. Unpaired Student's t-test values are compared to Mock condition unless otherwise stated.

Knowing that Poly(I:C) HMW is a strong but non-specific activator of TLR3, while Poly(A:U) HMW is a TLR3-specific agonist but with limited potency [28], we hypothesized that juxtaposing (I:C) and (A:U) blocks in 70 bp dsRNAs could combine the properties of both. We designed three chimeric molecules, dubbed TL-532, TL-533, and TL-534 (Supplementary Table S2 and **Fig. 2A**), in addition to a 70 bp Poly(I:C) (TL-535). Native PAGE electrophoresis showed that all molecules migrated as single bands of the expected sizes (Supplementary Fig. S2A). While the four novel 70 bp dsRNAs (TL-532 to TL-535) were more effective than TL-432 in reducing NCI-H292 cell viability (**Fig. 2C** and Supplementary Fig. S2C), only TL-532 and TL-533, which contain a Poly(A:U) track of at least 35 bp, induced higher level of inflammation compared to TL-432 and retained the ability to trigger the highest mTNFα secretion by RAW264.7s contrary to TL-534 and TL-535 (**Fig. 2B** and Supplementary Fig. S2B). To refine the relationship between Poly(A:U) and Poly(I:C) track length on biological response, we tested five additional ligands that are 70 bp TL-532 derivatives made of progressive replacement of (A:U) by (I:C) (TL-537 to TL-541) (**Fig. 2D**). We observed that a 70 bp dsRNA containing at least a 30 bp block of Poly(A:U) was necessary and sufficient to trigger both inflammatory cell death (i.e., accompanied by hIL-6 secretion) of NCI-H292s and mTNFα secretion by RAW264.7s (**Figs. 2 E-H**). Again, all effects disappeared in RAW264.7s when (I:C) greatly outnumbered (A:U) (TL-540 and TL-541) (**Fig. 2E**). Remarkably, the lack of activity of a 70 bp dsRNA with a random distribution of A-U-I-C nucleotides on both strands (TL-536) confirmed the importance of the (A:U) track for activation (**Figs. 2 E-H**).

Overall, our results indicate that chimeric 70 bp dsRNAs made of (I:C) and (A:U) blocks must contain Poly(A:U) tracks longer than 30 bp to efficiently trigger both myeloid cell activation and inflammatory apoptosis in epithelial cancer cells. Based on these data, we selected TL-532 for further study, and decided to abandon TL-533 as this latter faced hurdles for reproducible manufacturing on solid-phase support.

### TL-532 is a dsRNA with a defined sequence and size

**Figure 3:**
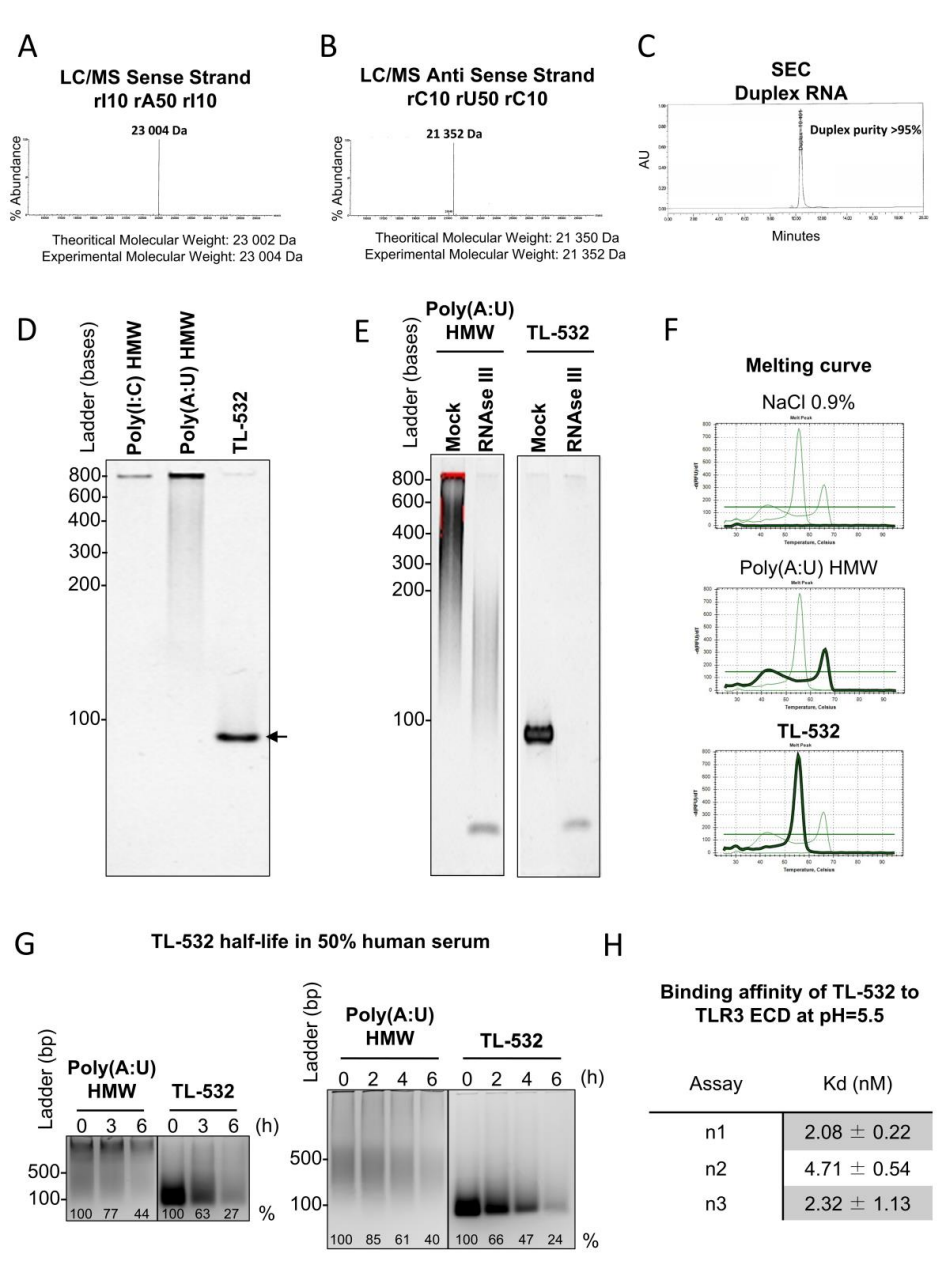
FIGURE 3: TL-532 is a well-defined dsRNA molecule. **(A-B)** Characterization of the sense strand 5′-rI(10)-rA(50)-rI(10)-3' and the antisense strand 5′-rC(10)-rU(50)-rC(10)-3' identities was performed by LC/MS. **(C)** The purity of the duplex RNA 5′-rI(10)-rA(50)-rI(10)-3' annealed with 5′-rC(10)-rU(50)-rC(10)-3' was evaluated by native SEC. **(D)** TL-532 70bp dsRNA was evaluated for its native gel profile in an 6% PAGE gel. 1 µg of each dsRNAs was deposited before visualization using bromide ethidium. **(E)** The double stranded RNA nature of TL-532 was evaluated by RNAse III limited digestion. 1 μg of each dsRNA was digested with 1 unit of RNAse III at 37°C for 10 min and deposited in a 6% native PAGE gel, before visualization using bromide ethidium. **(F)** The melting curve of TL-532 was evaluated using a Q-PCR melting curve program as described in Material and Methods section. **(G)** The half-life of TL-532 was evaluated in human serum. 2 μg of each dsRNA was incubated for increasing time in 50% human serum at 37°C and deposited in 2.5% agarose gel, before visualization using bromide ethidium. % = percentage of band intensity over the unincubated condition (0h). **(H)** Binding affinity of TL-532 to TLR3 Extra Cellular Domain (ECD) was determined by SPRi at pH=5.5. Data are representative of at least two (G) or three independent assays (A-F, H). Legend: LC/MS = Liquid Chromatography Mass Spectrometry; SEC = Size Exclusion Chromatography; SPRi = Surface Plasmon Resonance imaging.

We verified the purity and quality of TL-532 preparations by confirming the identity of both strands by LC/MS. We then confirmed the high annealed duplex purity above 95% by Size Exclusion Chromatography (**Figs. 3 A-C**). Consequently, TL-532 duplex migrated as one single homogenous band in native PAGE (**Fig. 3D**). The dsRNA nature of TL-532 was also confirmed by its complete sensitivity to the dsRNA-specific RNAse III digestion as well as by melting curve analysis showing a single peak around 56°C (**Figs. 3 E-F**), contrary to the heterogeneous profile of the Poly(A:U) HMW. TL-532 possessed an approximate four hours half-life in human serum (**Fig. 3G**) that is slightly lower than the estimated half-life of Poly(A:U) HMW (about five hours). SPRi analysis demonstrated that the affinity of TL-532 for the TLR3 Extra Cellular Domain is high (about 5 nM) at pH = 5.5 (**Fig. 3H**), corresponding to the acidic environment of endosomes where TLR3 signals [5, 31].

These experiments demonstrate that TL-532 is a dsRNA of 70 bp having a strong affinity for its TLR3 cellular target.

### TL-532 is a specific TLR3 agonist and does not activate other nucleic-acid sensors

**Figure 4:**
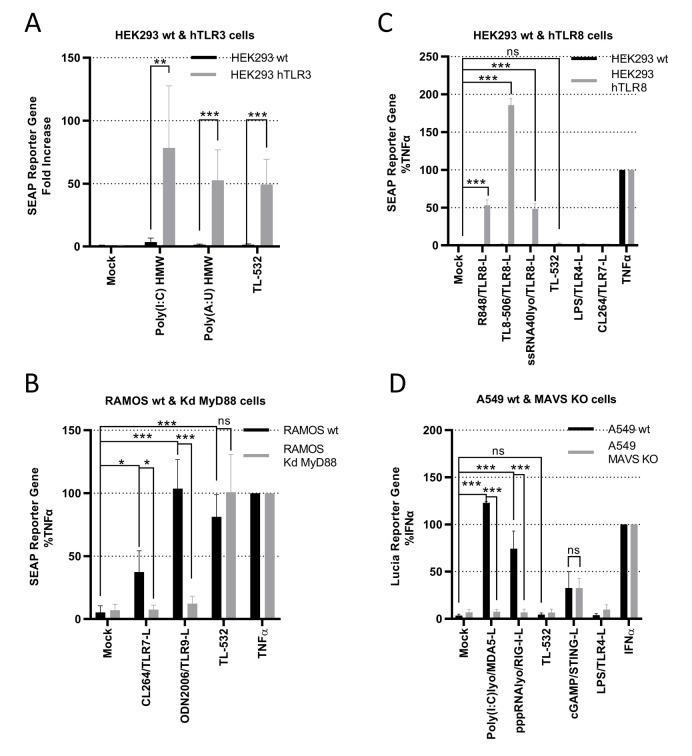
FIGURE 4: TL-532 is a specific agonist of TLR3 and does not activate TLR7-8-9, RIG-I, and MDA5. All the wt and modified cell lines used are stably transfected with either SEAP-reporter gene under control of NF-κB promoter or Lucia-reporter gene under control of ISRE promotor. **(A)** To confirm the capacity of TL-532 to activate TLR3, wild-type HEK293-Dual, that do not express TLRs (HEK293 wt), and its re-expressed human TLR3 counterpart (HEK293 hTLR3), were treated with 100 µg/mL of the indicated TLR3-ligand for 24 hours. NF-κB activation was measured with SEAP reporter gene assay and expressed as fold increase of untreated cells. **(B)** In order to analyze TL-532 specificity with regards to the TLR7 and TLR9, TL-532 activity was compared in RAMOS wt versus RAMOS MyD88 KD (the common adaptor of all TLRs except for TLR3 that signals only through TRIF). Cells were treated with 10 µg/mL of TLR7-ligand (CL264), 5 µg/mL of TLR9-ligand (ODN2006), 10 µg/mL of TL-532, and 0.1 μg/mL of TNFα for 24 hours. NF-κB activation was measured with SEAP reporter gene assay and expressed as the percentage of SEAP over their relative TNFα activation. **(C)** To analyze TL-532 specificity with regards to the TLR8, HEK293-Blue wt was reexpressed with human TLR8 (HEK293 hTLR8). Cells were treated with the three TLR8-ligands R848 at 1 µg/mL - TL8-506 at 1 µg/mL - ssRNA Lyovec at 5 µg/mL, TL-532 at 2000 µg/mL, TLR4-ligand LPS at 0.1 µg/mL, TLR7-ligand CL-264 at 5 µg/mL, and 0.1 μg/mL of TNFα for 24 hours. NF-κB activation was measured with SEAP reporter gene assay and expressed as the percentage of SEAP over their relative TNFα activation. **(D)** To determine the TL-532 specificity with regards to MAVS-dependent cytosolic dsRNA helicases sensors RIG-I and MDA5, wild-type A549 cell line (A549 wt), known to express cytosolic sensors but weak TLR3 protein, has been knocked-out for the MAVS adaptor (A549 MAVS KO). Cells were treated with either 0.1 µg/mL of MDA5-ligand (Poly(I:C) HMW Lyovec – Poly(I:C)lyo), 1 µg/mL of RIG-ligand (pppRNA Lyovec - pppRNAlyo), 2000 µg/mL of TL-532, 30 µg/mL of STING-ligand (2'3'cGAMP), 0.1 µg/mL of TLR4-ligand (LPS), and 1000 IU/mL of IFNα for 24 hours. ISRE activation was measured with Lucia reporter gene assay and expressed as the percentage of Lucia over their relative IFNα activation. Data are the mean of three independent experiments. Results are expressed as mean ± SD. Unpaired Student's t-test: * = p<0.05; ** = p<0.01; *** = p<0.001; ns: not significant.

We used several *in vitro* assays to demonstrate TL-532 specificity for TLR3. First, TL-532 activated HEK293 cells overexpressing hTLR3 (Supplementary Fig. S3A) as did the two positive controls Poly(I:C) HMW and Poly(A:U) HMW, but not the parental cell line that does not express hTLR3 (**Fig. 4A**).

Second, we determined whether TL-532 could activate endosomal TLR7 or TLR9 in RAMOS human B cells defective for MyD88, the adaptator for these two receptors. As RAMOS cell line is known to express TLR3 [34] and this knockdown (Kd) MyD88 model was validated by others [35], we confirmed the expression of TLR7 and TLR9 in the RAMOS wt and MyD88-Kd (Supplementary Fig.S3B-S3D). TL-532 activated NF-κB at similar levels in both RAMOS wild type (wt) human B cells and their MyD88-Kd derivatives, while CL264 (TLR7 agonist) and ODN2006 (TLR9 agonist) were only active in the wt cells (**Fig. 4B**), demonstrating that TL-532 does not activate these two receptors. Moreover, we showed that TL-532-induced biological response is abolished in RAMOS wt cells in presence of the H^+^ V-ATPase inhibitor Bafilomycin A1, an inhibitor of endosomal TLRs recognizing RNA including TLR3 [17, 36], reinforcing that TL-532 mainly activates TLR3 in this cell line (Supplementary Fig. S5A).

Third, we checked whether TL-532 could activate TLR8 using HEK293s overexpressing hTLR8 (HEK hTLR8) as confirmed by Western Blot (**Fig. 4C** and Supplementary Fig. S3C). As expected, while positive controls (R848, TL8-506, and ssRNA LyoVec) activated NF-κB in HEK-hTLR8s but not in wt cells, TL-532 did not activate TLR8 in HEK-hTLR8s as did the negative controls lipopolysaccharide (LPS) and CL264.

Finally, we next evaluated whether TL-532 activates the cytosolic sensors RIG-I and MDA5. Therefore, RAW264.7s were pretreated with pan-cathepsin inhibitor Z-FA-FMK [5] or H^+^ V-ATPase inhibitor Bafilomycin A1 [36], two drugs that inhibit endosomal TLR signaling (Supplementary Figs. S5B and S5C). As expected, LPS-induced mTNFα secretion was not inhibited by these drugs, demonstrating that MyD88-dependent cytoplasmic transduction signaling pathway was not disrupted. On the contrary, Z-FA-FMK and Bafilomycin A1 inhibited the activity of TL-532, demonstrating that TL-532 is strictly dependent on endosomal signaling where TLR3 initiates its transduction signaling pathway [5], and does not activate cytosolic sensors. RIG-I/MDA5 activation was also studied in A549 wt human epithelial lung cancer cells (**Fig. 4D**) that express very low levels of TLR3 (Supplementary Fig. S4A), but express RIG-I and MDA5 (Supplementary Fig. S4B) at similar levels compared to the NCI-H292 and at higher levels compared to the THP-1 control cells [14, 37]. TL-532 did not activate the ISRE reporter gene in wt cells unlike LyoVec-transfected Poly(I:C) HMW and pppRNA positive controls (ligands for MDA5 and RIG-I, respectively). These controls induced a strong biological response in A549s wt, but not in A549s lacking MAVS (the adaptor protein for RIG-I and MDA5); the lack of expression in this model was confirmed by others [38]. A549 cells do not express TLR4 and were not activated by LPS. STING activation was similar in A549 wt and MAVS knockout (KO), showing that MAVS-independent signaling pathways are functional and behave the same in the two cell lines. These data show that TL-532 does not activate MAVS-dependent cytosolic sensors.

All together, these experiments demonstrate that TL-532 activity proceeds exclusively through TLR3 and does not depend on TLR7, TLR8, TLR9, RIG-I, or MDA-5.

### TL-532 induces TLR3-dependent inflammatory and immunological cell death in NCI-H292 cancer cell, and anti-tumoral soluble factors secretion in RAW264.7 cells

**Figure 5:**
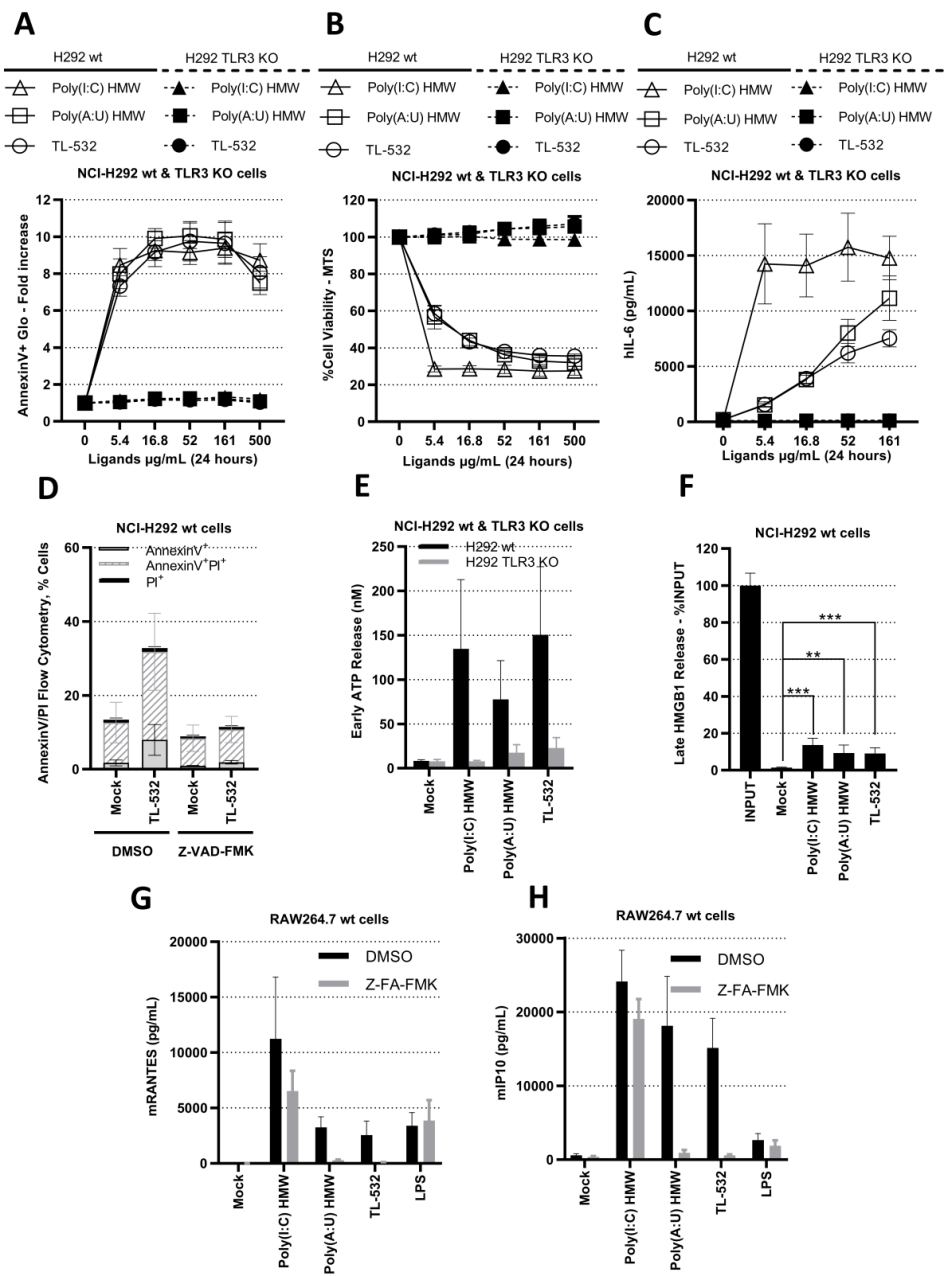
FIGURE 5: TL-532 induces both TLR3-dependent inflammatory/immunogenic cell death by apoptosis in NCI-H292 wt cancer cells and mRANTES/mIP10 secretion in RAW264.7 wt. **(A-C)** NCI-H292 wt cells and/or its TLR3 KO counterpart were treated with TLR3-ligands at indicated doses for 24 hours. TLR3-agonist activity was determined: by the fold apoptosis compared to untreated cell lines using AnnexinV-Glo assay **(A)**, Cell viability by MTS assay **(B)**, and cytokine secretion by hIL6-ELISA **(C)**. **(D)** The apoptotic nature of cell death in NCI-H292 wt cells was determined by adding or not the pan-caspase inhibitor Z-VAD-FMK (20 µM for 2 hours) prior to TL-532 treatment at 100 µg/mL for 6 hours. Percentage of AnnexinV+/Pi+ cells was analyzed by flow cytometry. **(E-F)** *In vitro* immunogenic cell death was determined in NCI-H292 cells by quantifying the following biomarkers in cell culture supernatant: ATP at 4 hours post-treatment using ATP-Glo assay (E) and HMGB1 by ELISA at 24h hours post treatment, expressed as the percentage of released HMGB1 compared to the total amount of intracellular HMGB1 (F). **(G-H)** RAW264.7 wt cells were pretreated with the pan-cathepsin inhibitor Z-FA-FMK (50 μM for 48hr) before treatment with 500 µg/mL of Poly(I:C) HMW and Poly(A:U) HMW and TL-532, and with 1 µg/mL of LPS, for 24 hours. The concentration of mRANTES (G) and mIP10 (H) was measured by ELISA. Data are the mean of three independent assays (A-H). Results are expressed as mean ± 95% confidence interval (A, B, C, E, G, H). Results are expressed as mean ± SD (D, F). Unpaired Student's t-test: * = p<0.05; ** = p<0.01; *** = p<0.001; ns: not significant.

Wt and TLR3 KO NCI-H292 cells were used to further characterize the direct anti-tumor effect of TL-532 (Supplementary Fig. S4C). TL-532, Poly(A:U) HMW, and Poly(I:C) HMW induced dose-dependent inflammatory cell death in NCI-H292 wt cells, that was abolished in NCI-H292 TLR3 KO cells, confirming the strict requirement for TLR3 in this cell line (**Figs. 5 A-C**) [14]. Reduced cell viability (≥30%) was also observed in two other cancer cell lines (Supplementary Figs. S6A-B). NCI-H292 wt cell death occurred through apoptosis, as shown by the Annexin V Glo assay and Annexin V/PI staining (**Fig. 5A** and Supplementary Figs. S6C-D). The apoptotic nature of cell death induced by TL-532 is confirmed by the complete absence of death after pretreatment with the pan-caspase inhibitor Z-VAD-FMK (whose efficacy was demonstrated by inhibiting caspase3/7 activation – see Supplementary Fig. S7), leading to a complete loss of AnnexinV+ Pi+ cell staining (**Fig. 5D**). The fact that NCI-H292 wt cells express low RIP3 protein levels [3] must explain the absence of necroptosis participation in this cell death, even when caspase-8 is compromised [14, 39]. In NCI-H292 wt cells, TL-532-induced apoptosis displays the hallmarks of immunogenic cell death [40], as shown by the early release of ATP (four hours post-treatment) and late release of HMGB1 (24 hours post-treatment; **Figs. 5 E-F**). As shown in Supplementary Figs. 8A–B, a single TL-532 treatment at 500 µg/mL for 24 hours was performed on *ex vivo* thick sections of fresh melanoma metastasis tumors from patients to conserve the tissue architecture and stroma. Under these conditions, pathologist found a significant increase of apoptotic bodies counts (2.5 fold) that correlated with both a significant decrease of tumor proliferation (Ki67+ staining) and a disruption of the tumor tissue. This demonstrates the anti-tumoral activity of TL-532 as a monotherapy on fresh *ex vivo* samples.

**Figure 6:**
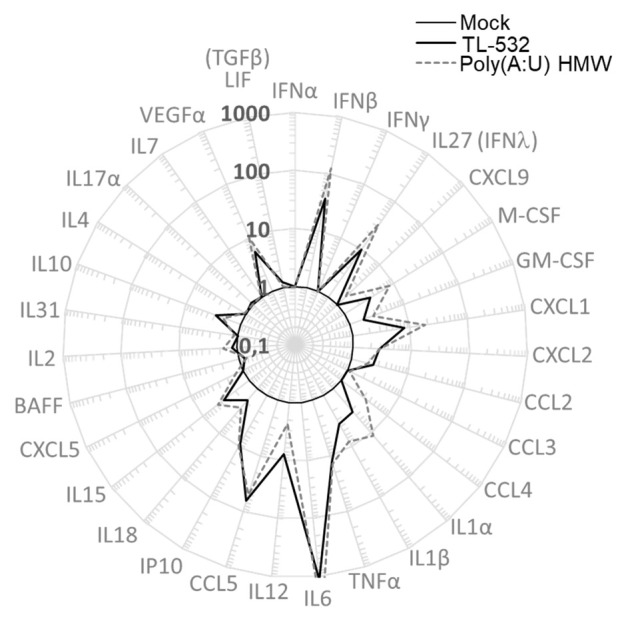
FIGURE 6: TL-532 and Poly(A:U) HMW induce the same profile of inflammation in RAW264.7 wt cells. RAW264.7 wt cells were treated with 500 µg/mL of Poly(A:U) HMW and TL-532 for 24 hours. The concentration of murine soluble factors was measured by Multiplex immunoassay. Results are expressed in fold increase compared to the Mock condition. Data are representative of two independent assays. LIF is a biomarker of TGFβ secretion; IL27 is a biomarker of IFNλ secretion.

Lastly, RAW264.7 wt cells were shown to secrete inflammatory factors such as IFNβ, IFNλ, mIP10 (CXCL10), and mRANTES (CCL5) under TL-532 treatment as efficiently as did Poly(A:U) HMW (**Fig. 6**). These cytokines/chemokines have been described to be associated with efficient antitumor immunity [41–43]. In contrast to the Poly(I:C) HMW known to activate RIG-I/MDA5 in addition to TLR3 in myeloid cells [28], the TLR3-specific Poly(A:U) HMW [28] and TL-532 cytokine secretion of mRANTES and mIP10 was abolished after Z-FA-FMK pretreatment (**Figs. 5 G-H**), reinforcing the involvement of TLR3 in this TL-532-induced antitumor profile.

Altogether, these results show that TL-532 induces TLR3-mediated inflammation in both myeloid and cancer cells, and TLR3-mediated immunogenic cell death through apoptosis in cancer cells.

### TL-532-induced apoptosis is specific to cancer cells

**Figure 7:**
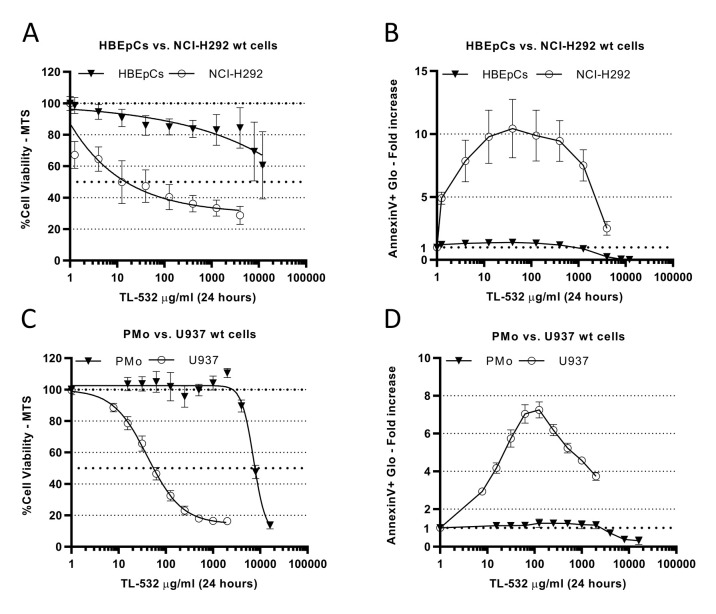
FIGURE 7: TL-532 induces apoptotic cell death in cancer cells but not in normal primary cell counterparts. Cell viability - expressed in percentage of untreated cells - was determined by MTS assay **(A, C)**, and apoptosis - expressed in fold increase compared to the mock control - was determined using AnnexinV-Glo assays **(B, D)**, after a 24 hr of dose escalation treatments of TL-532. **(A-B)** TL-532 induces apoptosis and reduces viability specifically in NCI-H292 wt lung cancer cells but not in the normal Human Bronchial Epithelial Primary Cells (HBEpCs). **(C-D)** TL-532 induces apoptosis and reduces viability specifically in U937 wt histiocytic lymphoma cancer cells but not in normal Primary Human Monocytes (PMo). All data are the means of three independent assays. Results are expressed as mean ± 95% confidence interval.

We next compared TL-532-induced apoptosis in two cancer cell lines and in corresponding primary cell lines: NCI-H292 wt pulmonary cancer cells vs. primary bronchial cells (HBEpCs) (**Figs. 7 A-B**) and acute myeloid leukemia cells (U937 wt) vs. primary monocytes (PMo; **Figs. 7 C-D**). The median toxic dose (TD50) of TL-532 on HBEpCs could not be reached. Consequently, comparison of TD25 to 25% maximum effective concentration (EC25) in NCI-H292s (4,000 vs. 2 µg/mL, respectively) revealed a 2000-fold difference of sensitivity between cancer and primary cells (**Fig. 7A**). In contrast to normal cells, tumor cell death occurred through apoptosis (**Fig. 7B**). A 140-fold sensitivity difference was observed when comparing the TD50 of TL-532 in PMos vs. its EC50 in U937s (8,000 vs. 57 µg/mL, respectively; **Fig.7C**). Again, tumor cell death occurred through apoptosis (**Fig. 7D**) in a TLR3 dependent manner as shown using Bafilomycin A1 (Supplementary Fig. S9).

Lack of toxicity was confirmed in HUVECs and PHHs (Supplementary Figs. S10A-B). These results indicate that TL-532 possesses anti-tumor effects and optimal tolerance in normal cells.

## DISCUSSION

In the present study, we identified a new family of TLR3 agonists that activates myeloid cells, triggers the secretion of pro-inflammatory cytokines in both myeloid and cancer cells, and induces apoptosis specifically in cancer cells. These novel molecules are TLR3-specific agonists that do not activate other RNA sensors. They induce both inflammatory apoptosis in cancer cells and activation of myeloid cells, and therefore represent useful tools to explore the role of TLR3 in cancer therapy. They do not require transfection agents, are chemically manufactured on solid-phase support, can be produced at large scale, and are perfectly defined in terms of sequence and size.

The screening based on inflammation and cell death highlighted that both length [4, 32] and sequence [33] are crucial for TLR3-dependent effects in RAW264.7 macrophages and in NCI-H292 epithelial lung cancer cells. Surprisingly, this stepwise approach revealed discrepancy between myeloid cell activation and apoptosis induction in cancer cells. (i) Among all 50 bp molecules tested, only homopolymeric poly(A:U) induced inflammation in RAW264.7s. Heteropolymeric poly(A:U-U:A) of the same length and base composition failed to activate RAW264.7s, demonstrating that dsRNA sequence impacts cellular response. (ii) Increasing the poly(A:U) sequence up to 70 bp correlated with increased apoptotic cell death in NCI-H292s without increasing potency in RAW264.7s. Random 70 bp sequences showed no activity. (iii) Further addition of homopolymeric Poly(I:C) blocks at the extremities of the 70 bp molecules was crucial for increasing inflammation in RAW264.7s and cell death in NCI-H292s. This rational discovery process led to the identification of TL-532 as lead molecule. While progressive replacement of (A:U) by (I:C) initially retained activity in the two cell lines, all effect disappeared in the RAW264.7 cells when (I:C) outnumbered (A:U), without interfering with NCI-H292 activation. These results clearly demonstrate that dsRNA of 70 bp must contain a minimal (A:U) content to be fully active. Moreover, these observations pave the way to better understand the different biological responses to improve TLR3 activation.

These differences in dsRNA activity could be related to TLR3 biology. TLR3 activation requires two steps. First, dsRNA is recognized by various uptake receptors including scavenger receptor class-A, Raftlin, or CD14, in a cell type-dependent and in a dsRNA length- and composition-dependent manner [1, 26, 44]. Second, dsRNA internalization occurs by endocytosis into TLR3^+^ endosomes to activate TLR3 [5, 45] with a minimal length of about 46 bp to support the homodimerization of at least two TLR3 receptors [4, 31], and dsRNA length has been shown to impact endolysosomal routing [32]. In this context, we hypothesize that length and (I:C) content of our molecules may positively influence both cellular entry and subcellular localization to deliver more material in TLR3^+^ endolysosomes. In fact, previous observations on TLR9 ligands have demonstrated differences in endosomal trafficking in early vs. late endosomes according to their structural features [46]. These findings may explain the different biological responses observed between the myeloid and the epithelial cancer cells in our hands. It may also explain why the 70 bp TL-532 containing (I:C) blocks has greater efficacy compared to its parental Poly(A:U) 70 bp molecule.

Our *in vitro* data clearly demonstrate that TL-532 induces TLR3-specific immunogenic cancer cell death via caspase-mediated apoptosis, and reduces tumor cell viability at similar level compared to Poly(A:U) HMW [15, 24] in all the tested cancer cells, while it is well tolerated in primary human cells leading to a wide therapeutic index. Although participation of necroptosis was not demonstrated in our experiments using TL-532, we cannot exclude an implication of this cell death in cancer cells that would express high levels of RIP3, a key protein responsible for necroptosis induction [39]. A direct TLR3-mediated cancer cell death is expected to be instrumental to get an anti-tumor response in humans as suggested by Salaun *et al.* [24]. These authors demonstrated in a retrospective clinical study that systemic administration of Poly(A:U) was associated with a significant decrease relapse in TLR3-positives but not in TLR3-negative primary cancers. In this context, new studies are needed to better understand the link between TLR3-induced cancer cell death and immune system activation. Resistance to TLR3-induced apoptosis in primary cells has been previously attributed to two molecular brakes, cIAP and cFLIP [3, 14], that are often released during tumorigenesis. In addition to the biological responses of TL-532 in cancer and normal cells, we also demonstrate that TL-532 induces anti-tumor cytokines/chemokines secretion in a TLR3-dependent manner in RAW264.7 cells. This response was similar to the results obtained with the Poly(A:U) HMW, which is a well-known TLR3 agonist that activates myeloid cells [13, 28]. Poly(A:U) HMW is a mixture of dsRNA and ssRNA of various lenght (150 to 8,000 bases pairs) leading to variable activity from batch-to-batch. This variation represents a hurdle to elucidate the role of TLR3 in chronic diseases and limits its potential clinical applications. Unlike Poly(A:U) HMW, the short 70 bp dsRNA TL-532 is chemically synthesized on solid-phase support in a reproducible manner which is an indispensable prerequisite to comply with GMPs (Good Manufacturing Practice) standards, and can be used as a true TLR3 agonist tool to deeply decipher the TLR3 pathway in oncology translational research. Regarding the capacity of TL-532 to induce TLR3-dependent inflammation and specific tumor-cell death, further animal studies will be necessary to confirm our *in vitro* observations.

Unlike TL-532, clinically tested Poly(I:C) derivatives such as Rintatolimod [27], Poly-ICLC [29, 47], and BO-112 [30] have ill-defined structure and size; the latter two are non-TLR3 specific cell death inducers and activate MDA5 among others, contributing to systemic toxicity [48]. Moreover, and in contrast to our results that show more than 100-fold differential sensitivity between normal and cancer cells, Besch *et al.* [49] reported that *in vitro* transfected poly(I:C) activates RIG-I and MDA5, leading to low dose toxicity. Rintatolimod (Poly(I:C_12_U)) is considered as an inducer of inflammation acting mainly through TLR3 [50, 51] but has not been reported to induce tumor cell death. Other TLR3 agonists are currently being evaluated at the preclinical stage. Recently, Koerner *et al.* showed that PLGA-particles carrying Riboxxim have adjuvant effects and induce tumor regression in syngenic murine cancer models expressing OVA when Riboxxim and the corresponding antigen are encapsulated together [37]. Riboxxol and its derivative Riboxxim are dsRNAs of 50 and 100 bp, respectively, made of randomly added rG and rI [52]. However, Riboxxol and its derivative Riboxxim contain an uncapped triphosphate moiety at the 5'end that is responsible for activating RIG-I in addition to TLR3 [37, 52]. In fact, systemic administration of RIG-I agonists may be associated with adverse events in human [53], and route of administration and doses of such compounds should be carefully evaluated to prevent unwanted activation of this cytosolic sensor. ARNAX is also a well-defined TLR3-specific agonist [54]. It is a sODN-140mers dsRNA, with RNA sequence chosen from the measle virus genome, and it is able to activate TLR3 without inducing inflammation. ARNAX with Tumor Associated Antigens (TAA) in combination with anti-PD-L1 antibody induces anti-tumor immunity and enhances tumor remission in mouse models [55]. It has not been reported if ARNAX induces direct cancer cell death. Lastly, Hyun Ko *et al.* [56] developed NexaVant (NVT), a novel dsRNA-based TLR3 agonist of 424 base pairs chosen from Chinese sacbrood virus (CSBV) genome as an effective vaccine adjuvant.

In conclusion, and in a growing field of innate immune agonists development in oncology, we conducted the rational design of a new family of perfectly defined TLR3 agonists. The identified lead molecule TL-532 is a dsRNA of 70 bp that has unique sequence and size, is manufacturable on solid-phase support and has strong affinity for its cellular target. TL-532 induces both *in vitro* specific cancer cell apoptosis without inducing toxicity in primary normal cells and *in vitro* RAW264.7 myeloid cell activation. Thus, TL-532 represents a novel tool to decipher TLR3 function, with potential to improve clinical landscape to fight cancer.

## MATERIALS AND METHODS

### Cell culture

Human non-small cell lung cancer NCI-H292 wt (Cat. CRL-1848; RRID: CVCL_0455), murine macrophage RAW264.7 wt (Cat. TIB-71; RRID:CVCL_0493), human histiocytic lymphoma U937 wt (Cat. CRL-1593.2; RRID:CVCL_0007), human pharyngeal carcinoma Detroit-562 wt (Cat. CCL-138; RRID:CVCL_1171), monocyte THP-1 wt (Cat. TIB-202; RRID:CVCL_0006) cells were purchased from ATCC (LGC Standards, France). TLR3-Knockout (TLR3 KO) NCI-H292 cells were generated and kindly provided by Pr. Lebecque (CRCL, Lyon, France). Human renal carcinoma TUHR14TKB wt (Cat. RCB1383; RRID:CVCL_5953) was purchased from RIKEN BioResource Center (Japan). NCI-H292 wt and TLR3 KO cells were grown in RPMI-1640 medium (Gibco, Thermo Fischer Scientific) supplemented with 10% decomplemented FBS, NaPy 1 mM (Gibco), 100U/mL penicillin/streptomycin (Gibco), and 2 mM Glutamine (Gibco). RAW264.7 cells were grown in DMEM media (Gibco) supplemented with 10% decomplemented FBS and 100 U/mL penicillin/streptomycin. U937 and THP-1 cells were grown in RPMI-1640 media (Gibco) supplemented with 10% decomplemented FBS and 100 U/mL penicillin/streptomycin. Detroit-562 cells were grown in DMEM media (Gibco) supplemented with 10% decomplemented FBS and 1% glutamine. TUHR14TKB cells were grown in RPMI-1640 media (Gibco) supplemented with 10% decomplemented FBS and 1% glutamine. HEK293-Dual (NF/IL8) wt (Cat. hkd-nullni) and hTLR3 (Cat. hkd-htlr3ni), HEK293-Blue wt (Cat. hkd-null1) and hTLR8 (Cat. hkd-htlr8), A549-Dual wt (Cat. a549d-nfis) and MAVS KO (Cat. a549d-komavs) (A549-Dual MAVS KO model was validated by others [38]), and RAMOS-Blue wt (Cat. rms-sp) and MyD88 Knockdown (MyD88 KD) (Cat. rms-kdmyd) (RAMOS-Blue MyD88 KD model was validated by others [35]) were purchased from Invivogen (France) and were grown according to the manufacturer's recommendations. These cell lines are known to express TLR3 [5, 18, 20, 34, 57] unlike HEK293-Dual [5], A549-Dual [58] and NCI-H292 KO (Supplementary Fig. S4C). RAMOS-Blue cells are known to express TLR7 and 9 in addition to TLR3, but does not express TLR8 (Technical Support Invivogen).

Human Bronchial Epithelial cells HBEpCs (Cell Applications, CA, USA) and Human Umbilical Vein Endothelial Cells (HUVECS; LGC Standards) were grown according to the manufacturer's recommendations. Human Primary Monocytes (PMo) were provided from EFS (Lyon, France) and were grown in RPMI-1640 supplemented with decomplemented 10% human serum (NeoBiotech, Nanterre, France), 100 IU/mL penicillin/streptomycin, and NaPy 1 mM. Primary Human Hepatocytes (PHH) were obtained from INSERM U1052 (Lyon, France) and were grown as described previously [59]. All cells were platted 24 hours before treatment at the following densities: 10^4^, 3x10^4^, or 5x10^5^ NCI-H292 wt and TLR3 KO cells in 96-wells, 48-wells, or 6-wells respectively; 5x10^4^ RAW264.7 cells in 96-wells; 2.5x10^4^ U937 cells in 96-wells; 1.2x10^4^ Detroit-562 cells in 96-wells; 0.75x10^4^ TUHR14TKB cells in 96-wells; 2.5x10^4^ or 5x10^5^ HEK293-Dual wt and hTLR3 cells in 96-wells or 6-wells respectively; 2x10^4^ HEK293-Blue wt and hTLR8 cells in 96-wells; 2.5x10^4^ or 5x10^5^ A549-Dual wt and MAVS KO cells in 96-wells or 6-wells respectively; 10^5^ RAMOS-Blue wt and Kd-MyD88 cells in 96-wells; 10^4^ HBEpCs in 96-wells; 7-10x10^4^ PMo in 96-wells; 7x10^4^ HUVECS in 96-wells; 1.25x10^5^ PHH in 96-wells.

### Reagents

Poly(I:C) High Molecular Weight (HMW), Poly(A:U) HMW, Poly(I:C) HMW LyoVec, pppRNA LyoVec, LPS, 2'3'cGAMP, CL264, ODN2006, TL8-506, ssRNA40 LyoVec, and Bafilomycin A1 were purchased from InvivoGen. Human TNFα was purchased from PeproTech (NJ, USA). Universal IFNα was purchased from PBL Assay Science (NJ, USA). Z-FA-FMK was purchased from Sigma-Aldrich (MO, USA). Z-VAD-FMK was purchased from R&D Systems (MN, USA). RNAse III was purchased from Thermo Fischer. Human serums were purchased from NeoBiotech (France).

Synthesis of dsRNAs was performed by different manufacturers: IDT (IA, USA), Horizon Discovery (UK), or NittoAVECIA (CA, USA). A 45 grams batch of TL-532 was synthesized by NittoAVECIA under non-GMP standards. Lyophilized powders were resuspended using apyrogenic, nuclease-free, sterile NaCl 0.9% (InvivoGen). All dsRNAs were used without transfection reagents unless otherwise stated. NaCl 0.9% was used as the Mock condition for all experiments unless stated differently in the text.

### Gel electrophoresis

Nucleic acids were resolved in TBE 1x PAGE gel (Sigma-Aldrich), or in TAE 1x agarose gel (Sigma-Aldrich). Single- and double-stranded RNA ladders were purchased from Thermo Fisher Scientific and NEB (MA, USA). Nucleic acid staining was performed with 1 μg/mL ethidium bromide (Sigma-Aldrich). Image acquisition and relative band intensity measurement were performed using a Gel Doc XR+ (Bio-Rad, CA, USA) driven by Image Lab software version 6.1.

### Spectrophotometric and luminescence readings

All colorimetric and luminescence assays were performed using a Tecan Infinite M200 PRO microplate reader (Tecan, Switzerland). Concentrations of human IL-6 (hIL-6), murine TNFα (mTNFα), murine RANTES (mRANTES CCL5), and murine IP10 (mIP10 CXCL10) in cell culture supernatants were measured using an ELISA cytokine enzyme immunoassay (BioLegend, CA, USA) and recorded at 450 nm. Detection of SEAP in cell culture supernatant by QUANTI-Blue (recorded at 620 nm) and/or detection of Lucia in cell supernatant by QUANTI-Luc (recorded using luminescence) were performed according to the manufacturer's recommendations (InvivoGen). Early ATP release (4 hr after treatment, recorded using luminescence) was measured in the cell culture supernatant using the CellTiter-Glo Luminescent Cell Viability Assay (Promega, WI, USA). HMGB1 release (24 hr after treatment, recorded at 450 nm) was measured in cell culture supernatants by ELISA (IBL, Germany). All readings were performed following the manufacturers' recommendations.

### Mouse Multiplex Immunoassay

Mouse Multiplex Immunoassay was performed on cell culture supernatant using the Procartaplex Multiplex Assay Mix&Match 31-plex, and recorded using the Luminex Bioplex MAGPIX according to the manufacturer's recommendations (Thermo Fischer Scientific).

### Quantification of cell survival and apoptosis

MTS cell survival was measured using the CellTiter 96 Aqueous Assay reagent (Promega, WI, USA). LDH Release was measured using the CytoTox 96 Non-Radioactive reagent (Promega). Plates were recorded at 490 nm and 690 nm (plate background). Cell survival was measured using Red Neutral staining as described previously [59]. Plates were recorded at 540 nm. Apoptosis was measured with the RealTime-Glo Annexin V Apoptosis Assay (Promega) or with AnnexinV-FITC and propidium iodide (PI) (BioLegend) before flow cytometry analysis (FACSCalibur, BD, NJ, USA) driven by BD CellQuest software (BD). All assays were performed according to manufacturers' instructions.

### Western blotting

Western blotting was performed as described in Bonnin *et al.* [2], with the following modifications. Proteins were transferred onto PVDF membranes using semi-dry Trans-Blot apparatus (Bio-Rad), anti-actin was from MP (clone C4, France; RRID:AB_2335127), anti-hTLR3 (clone D10F10; RRID:AB_10829166; MA, USA), anti-hTLR7 (clone D7; RRID:AB_10692895), anti-hTLR8 (clone D3Z6J; RRID:AB_2797755), anti-hTLR9 (clone D9M9H; RRID:AB_2798290), anti-RIG-I (clone D14G6; RRID:AB_2269233), anti-MDA5 (clone D74E4; RRID:AB_10694490) were from Cell Signaling, and secondary Goat anti-Rabbit HRP (RRID:AB_430833) conjugate and secondary Goat anti-Mouse HRP (RRID:AB_430834) conjugate were from Promega. Image acquisition and relative band intensity measurement were performed using a Chemi Doc XR+ apparatus (Bio-Rad) driven by Image Lab software version 6.1.

### Liquid Chromatography-Mass Spectrometry (LC/MS)

2 μL of each RNA strand (1 mg/mL in water) were injected into an Agilent 1260 Infinity II with Agilent SQ MSD LIMS 2344. Mobile phase A was prepared with 2.0% 1,1,1,3,3,3-Hexafluoro-2-propanol and 0.2% hexylamine in water. Mobile phase B was prepared with 50% acetonitrile and 50% methanol. Gradient %A over %B was applied (0min 90%A; 20min 65%A; 35min 56%A; 36min 50%A; 37min 90%A; 42min 90%A) at a flow rate of 0.3 mL/min. For MS (Avecia), an Agilent single quad system or equivalent was used and recorded in negative ion mode.

### Size Exclusion Chromatography (SEC)

10 μL of duplex TL-532 (1 mg/mL in PBS) was injected into an Agilent 1200 Infinity LIMS 2048. Mobile phase was 3X PBS at a flow rate of 0.7 mL/min for 20 min at isocratic gradient.

### Melting curve analysis

1 μg of dsRNA were added in 1X Universal SYBR Supermix (Bio-Rad) and subjected to melting curve analysis (temperature decreasing by 5°C every 5 seconds before acquisition, from 95 to 25°C) using a CFX Connect Real-Time System (Bio-Rad).

### Surface Plasmon Resonance imaging (SPRi)

All buffers were treated with DEPC. Surface plasmon resonance imaging (SPRi) experiments were performed using the OpenPlex instrument from Horiba. Biochips, glass prism coated with a gold thin layer from Horiba were treated with carboxylated K-One surface chemistry from Kimialys (Paris, France). Surfaces were activated using EDC/NHS (EDC: 0.4 M N-[3-Dimethyl-aminopropyl]-N-ethylcarbodiimide hydrochloride; NHS: 0.1 M N-Hydroxysuccinimide, Cytiva) for 15 min prior to the immobilization of the amine-modified nucleic acids (TL-532 and a 40-base pair DNA, dsDNA). TL-532 and the dsDNA were diluted in the immobilization buffer provided by Kimialys to 50 µM and 40 µM, respectively, and spotted on the surface in a microarray format for 15 min. The biochips were then placed directly in the SPRi instrument under a continuous flow of the running buffer (Pipes-buffered saline (PiBS) [20 mM Pipes, 150 mM NaCl, pH 5.5]) at a flow rate of 50 μl/min. 1 M ethanolamine-HCl, pH 8.5 (Cytiva) was injected to block remaining active carboxyl groups for 10 min. TLR3 protein (R&D Systems) diluted in the running buffer at concentrations ranging from 0 to 50 nM was injected over the biochip surface for 180 s or 240 s and left to dissociate for 20 min. The surface was regenerated after each injection with a PiBS buffer at pH 7.5. To confirm the repeatability, measurements were performed on three different biochips.

The obtained data were analysed to extract the affinity (KD). The background signals were subtracted from the signal recorded at the TL-532 and dsDNA spots. The maximum response, measured at equilibrium (at the end of the association step), were plotted in the form of a Langmuir binding isotherm and fitted to determine KD. KD was calculated independently for each spot.

### TL-532 activity in freshly resected human tumors cultured *ex-vivo*

Informed consent was obtained from patient with vaginal metastasis with bladder invasion from melanoma. Tumor samples were obtained from Trans-Urethral Resection Bladder Tumors (TURBT). Freshly resected tumors were sliced in sections of 250 μm using a vibratome (Thermo Scientific Microm HM650V). Tumors sections were cultured in 1 ml of synthetic BEC-GM medium (Cell Applications), either in presence of TL-532 (500 μg/ml) or NaCl 0.9% for 24-48h at 37°C and 5% CO_2_, before being fixed in 4% formalin and embedded in paraffin. Paraffin sections of 4 μm were generated for morphological staining (Hematoxylin-Phloxine-Saffron) and pathological analyzes. Apoptotic bodies were quantified from the whole sample area (25-35mm^2^). TLR3 (Promokine, Rabbit Polyclonal Antibody, Cat. PKAB7183643) and Ki67 (Roche, Rabbit Monoclonal Antibody clone 30-9, Cat. 790-4286; RRID: AB_2631262) immunohistochemistry staining were performed as described in [2, 3].

### Statistics

All statistics were performed using GraphPad Prism 9 software (GraphPad Prism, CA, USA; RRID:SCR_002798). As mentioned in each figure, statistics were performed using either two-tailed unpaired or paired *t*-tests and was defined significant (*=*p*<0.05 **=*p*<0.01 ***=*p*<0.001; ns=not significant); or confident interval 95%.

## AUTHOR CONTRIBUTION

ST: Conceptualization, methodology, validation, formal analysis, investigation, writing original draft – review & editing. SM: Methodology, validation, formal analysis, investigation. AB: Validation, formal analysis, investigation. LD: Formal analysis, investigation. CP: Formal analysis, investigation. NV: Formal analysis, investigation. SO: Formal analysis, investigation, resources. MS: Formal analysis, investigation. SC: Formal analysis, investigation. MBJ: Formal analysis, investigation. MC: Resources. BW: Writing original draft – review & editing, supervision, project administration. MB: Conceptualization, methodology, validation, formal analysis, investigation, writing original draft – review & editing, supervision.

## SUPPLEMENTAL MATERIAL

Click here for supplemental data file.

All supplemental data for this article are available online at www.microbialcell.com/researcharticles/2023a-thierry-microbial-cell/.

## Abbreviations:

dsRNA: – double-stranded RNA,
HMW: – high molecular weight,
hIL: – human interleukin,
KO: – knockout,
LPS: – lipopolysaccharide,
mTNFα: – murine TNFα,
PMo: – primary monocytes,
TLR: – Toll-like receptor,
wt: – wild type.
