# Supplementary material for: TL-532, a novel specific Toll-like receptor 3 agonist rationally designed for targeting cancers: discovery process and biological characterization
Source: Microb Cell. 2023 Apr 19;10(6):117–32. doi: 10.15698/mic2023.06.797 (PMC10236204; doi:10.15698/mic2023.06.797)
Supplement: Supplementary file 1 [file mic-10-117-s01.pdf]

Supplementary Figure S1

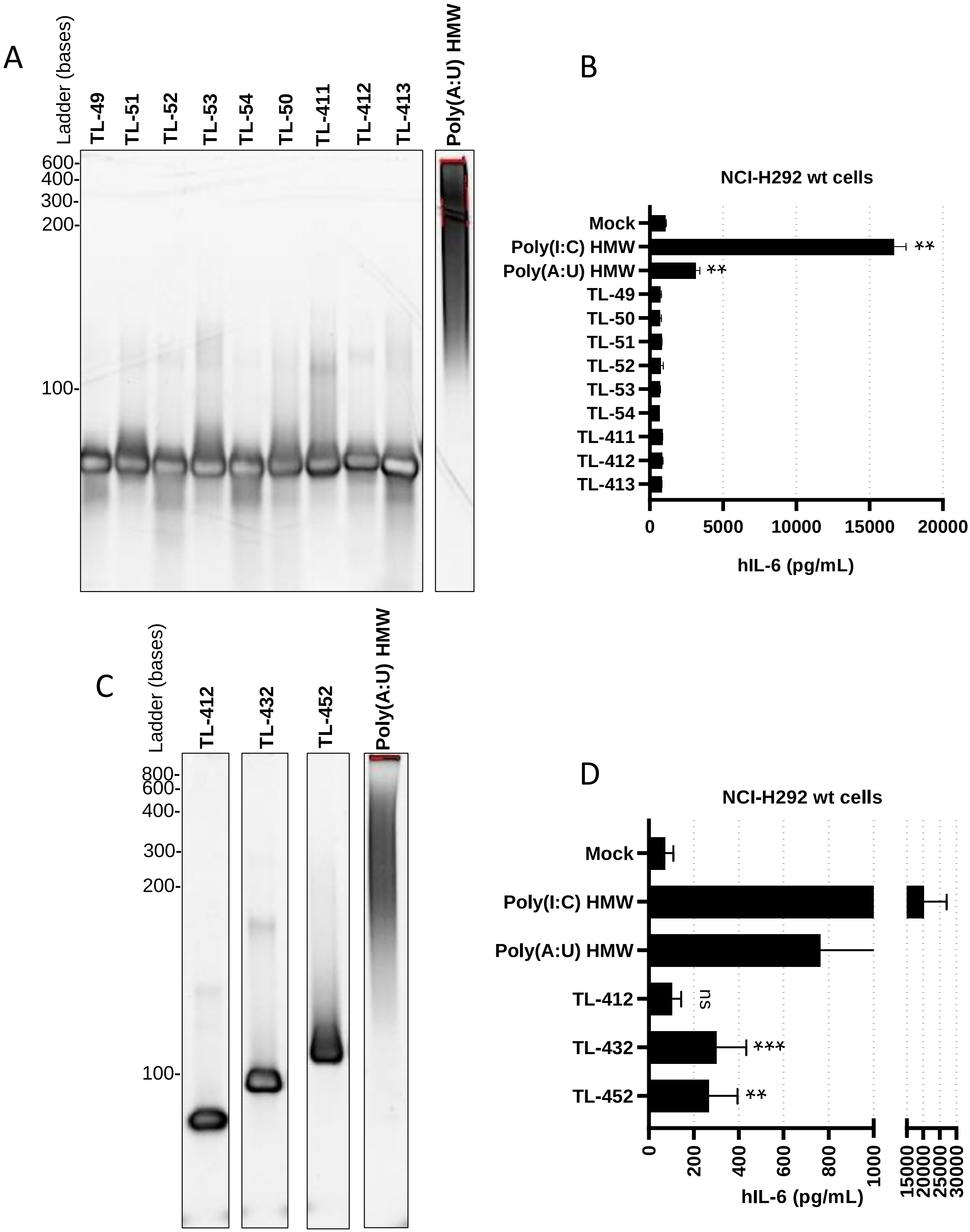

Supplementary Figure S1: Supporting data of the discovery process. (A) A panel of 50 bp dsRNAs were evaluated for their native gel profile in an 8% PAGE gel. 5 µg of each dsRNAs were deposited before visualization using bromide ethidium. **(C)** TL-412, TL-432, and TL-452 dsRNAs of respectively 50-70-90bp were evaluated for their native gel profile in an 6% PAGE gel. 1 µg of each dsRNA was deposited before visualization using bromide ethidium. **(B-D)** NCI-H292 wt cells were treated with the different molecules at 10 µg/mL for 24 hours. The concentration of hIL-6 was measured by ELISA. Data are representative of at least two (A) or three (B-C) independent assays, or are the mean of three independent assays (D). Results are expressed as mean ± SD. Unpaired Student's *t*-test: * = $p<0.05$; ** = $p<0.01$; *** = $p<0.001$; ns: not significant. Unpaired Student's *t*-test values are compared to Mock condition unless otherwise stated.

Supplementary Figure S2

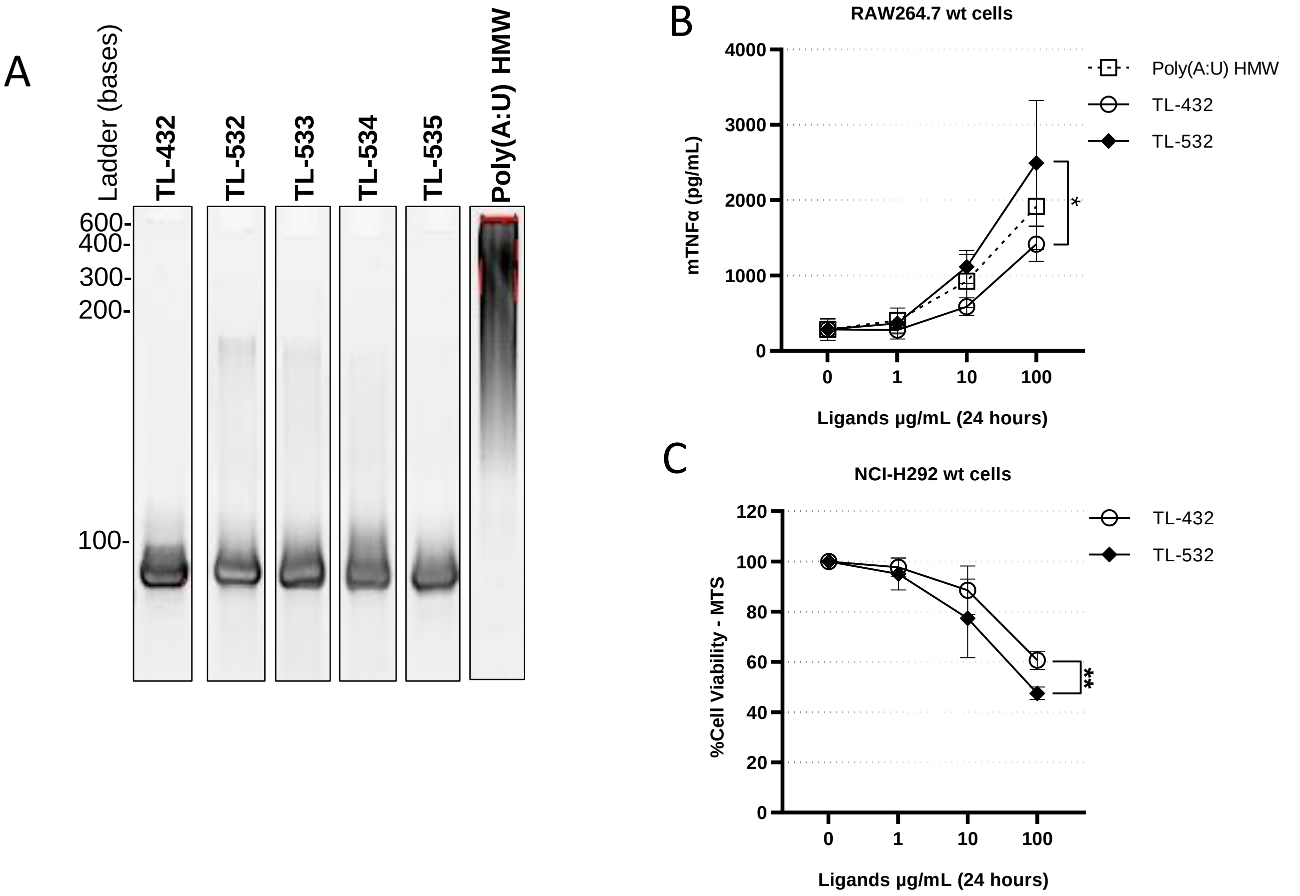

Supplementary Figure S2: Supporting data of the discovery process. (A) All 70bp dsRNAs TL-432, TL-532 thru -535 were evaluated for their native gel profile in an 6% PAGE gel. 1.5 µg of each dsRNA was deposited before visualization using bromide ethidium. **(B)** RAW264.7 wt cells were treated with the different molecules from 1 to 100 µg/mL for 24 hours. The concentration of mTNFα was measured by ELISA. **(C)** NCI-H292 wt cells were treated with the different molecules from 1 to 100 µg/mL for 24 hours. The cell viability was determined by MTS assay. Data are representative of at least two independent assays (A) or are the means of at least two (B) or three (C) independent assays. Results are expressed as mean ± SD. Unpaired Student’s *t*-test: * = $p<0.05$; ** = $p<0.01$; *** = $p<0.001$; ns: not significant.

Supplementary Figure S3

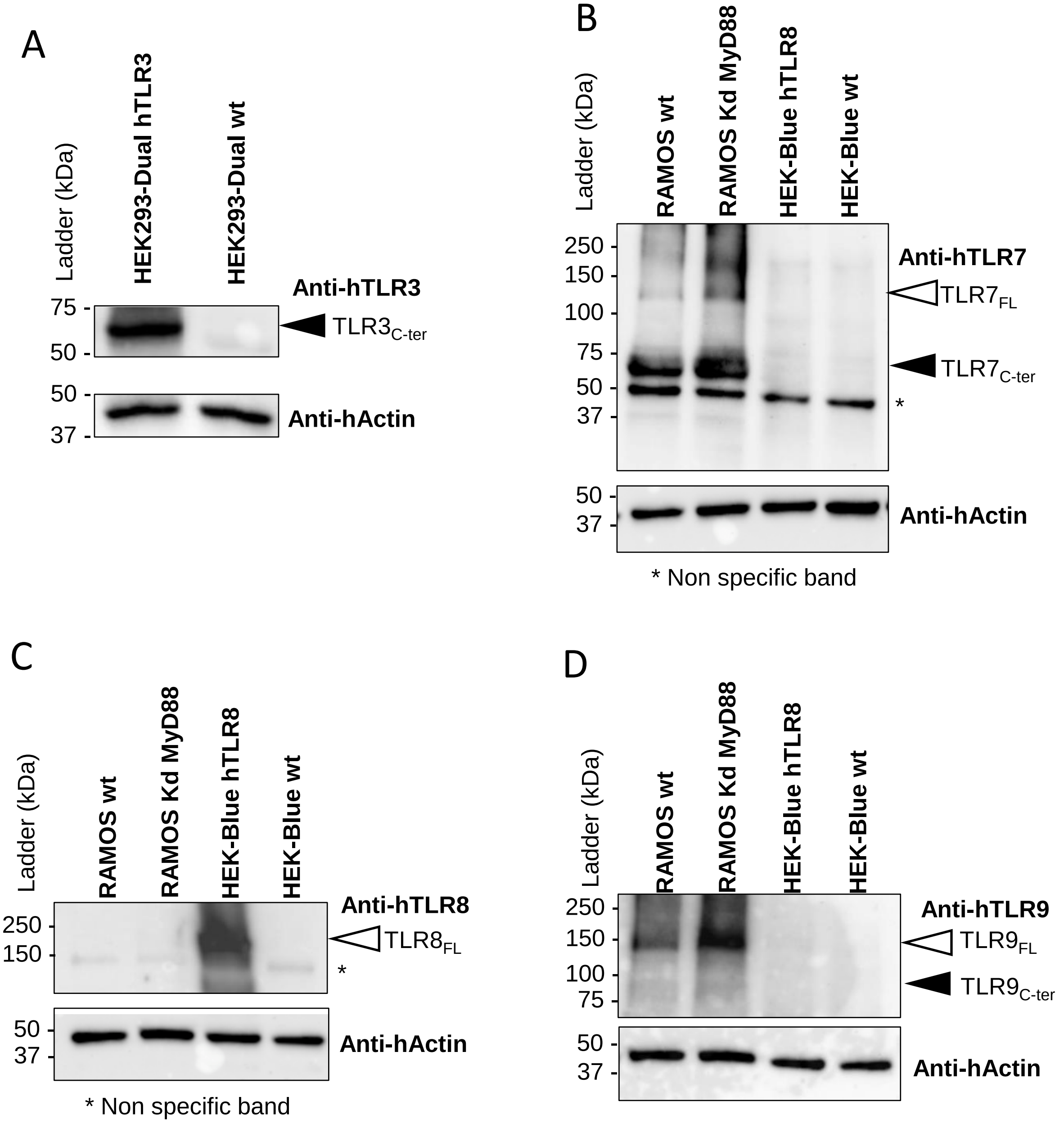

Supplementary Figure S3: Supporting data of the TLR3 specificity experiments of TL-532. (A) The TLR3 protein expression was evaluated by Western Blot in HEK293-Dual wt and its re-expressed human-TLR3 counterpart (HEK293-Dual hTLR3). $TLR3_{C\text{-}ter}$ = 72kDa cleaved C-ter band of TLR3 indicating that the receptor is localized in the endolysosomes. **(B)** The TLR7 protein expression was evaluated by Western Blot in RAMOS wt and Kd MyD88, compared to the TLR7 negative HEK293-Blue wt cells. $TLR7_{FL}$ = TLR7 Full Length; $TLR7_{C\text{-}ter}$ = 75kDa cleaved C-ter band of TLR7 indicating that the receptor is localized in the acidic compartments (Hipp et al., Immunity, 2013). **(C)** The TLR8 protein expression was evaluated by Western Blot in HEK293-Blue wt and its re-expressed human-TLR8 counterpart (HEK293-Blue hTLR8). **(D)** The TLR9 protein expression was evaluated by Western Blot in RAMOS wt and Kd MyD88, compared to the TLR9 negative HEK293-Blue wt cells. $TLR9_{FL}$ = TLR9 Full Length; $TLR9_{C\text{-}ter}$ = 80kDa cleaved C-ter band of TLR9 indicating that the receptor is localized in the endolysosomes (Ewald et al., Nature, 2008). Data are representative of two independent assays.

Supplementary Figure S4

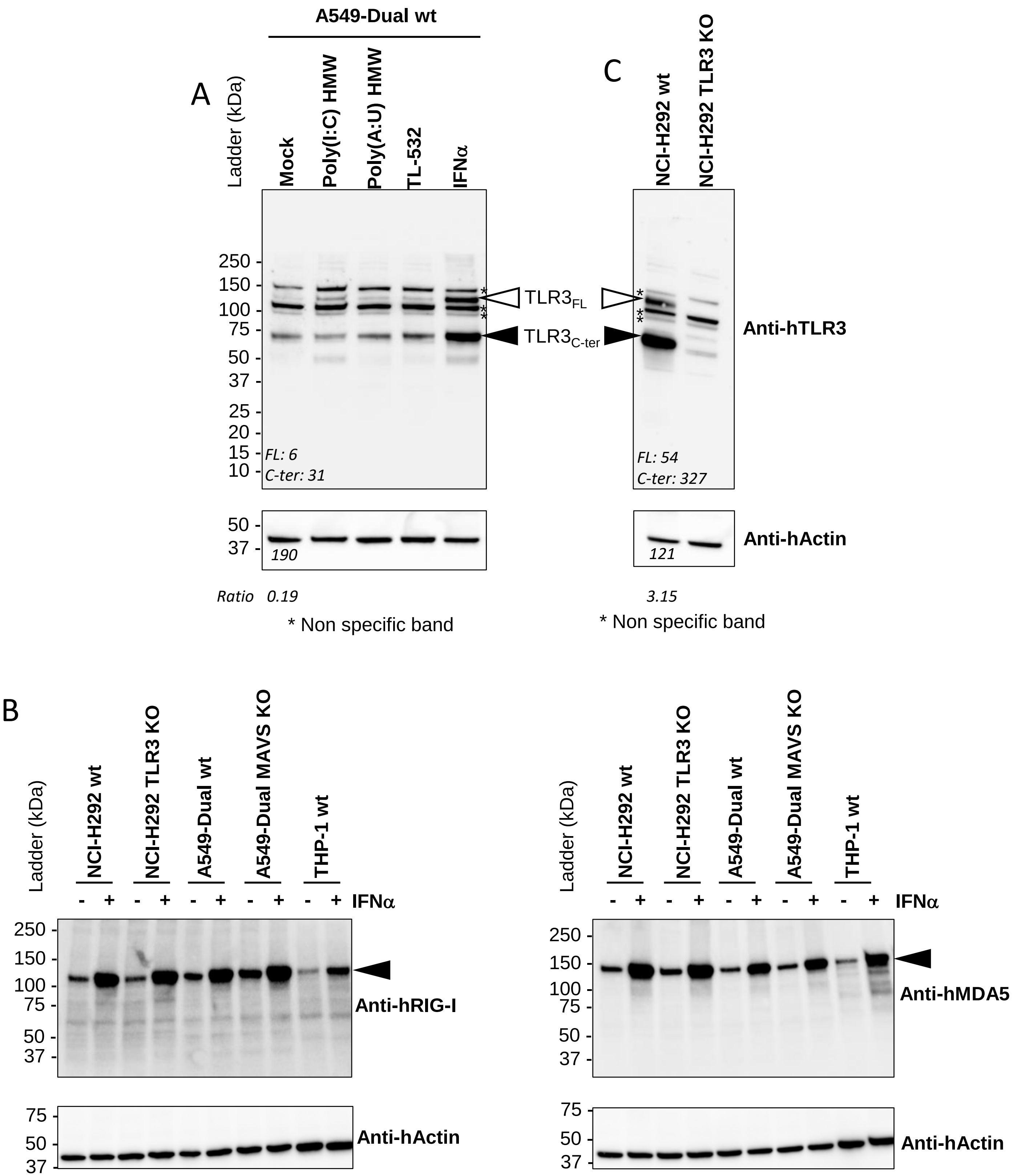

Supplementary Figure S4: Supporting data of the TLR3 specificity experiments of TL-532. (A-C) The TLR3 protein expression was evaluated by Western Blot in (A) A549-Dual wt cells at resting state or after 24 hours of treatment with 500 µg/mL of the indicated dsRNA or 1000 IU/mL of IFNα, (C) NCI-H292 wt (express endogenous hTLR3) and TLR3 KO H292 cells, confirming the absence of TLR3 expression in the KO counterpart. Western Blot legends: $TLR3_{FL}$ = TLR3 Full Length; $TLR3_{C\text{-}ter}$ = 72kDa cleaved C-ter band of TLR3 indicating that the receptor is localized in the endolysosomes. 6 = relative band intensity. Ratio is calculated according to the following formula: $(RelativeBandIntensity_{FL} + RelativeBandIntensity_{Cter\text{-}TLR3}) / RelativeBandIntensity_{Actin}$. * = Non-specific band. **(B)** The RIG-I and MDA5 protein expression were evaluated by Western Blot in A549-Dual wt and MAVS KO cells at resting state or after 16 hours of treatment with 1000 IU/mL of IFNα. The RIG-I and MDA5 expression levels of A549 were compared to the NCI-H292 and THP-1 wt cells known to express these two cytosolic sensors (Estornes et al., CDD, 2012; Koerner et al., Nat Com, 2021). Data are representative of two independent assays.

Supplementary Figure S5

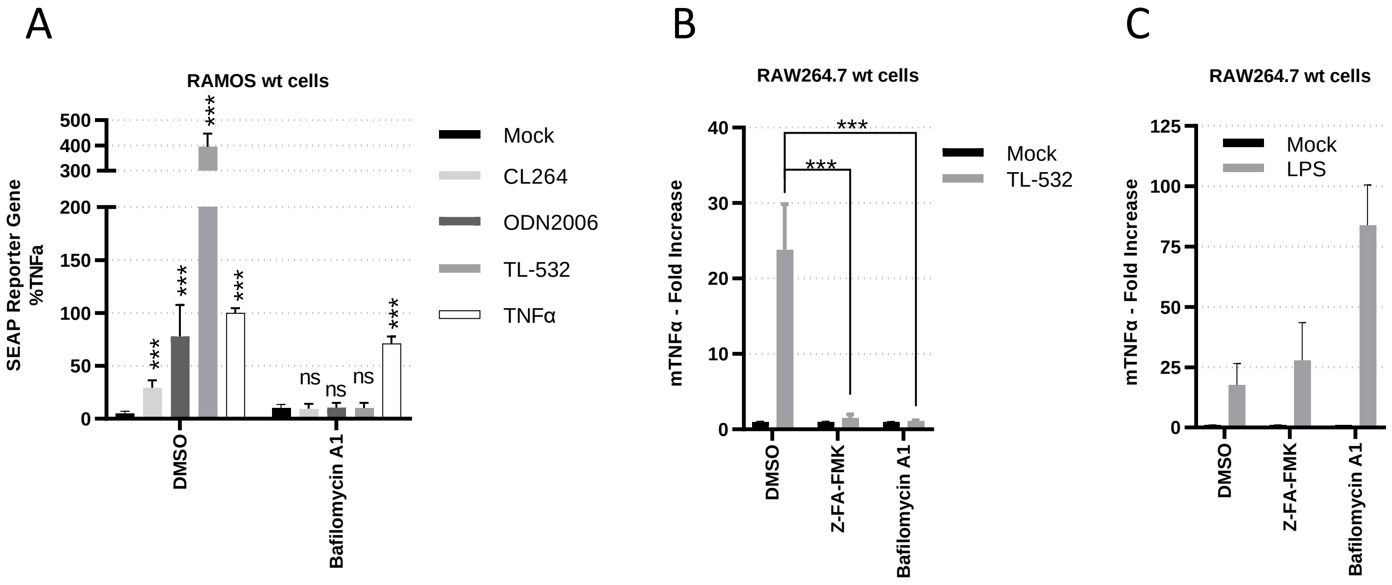

Supplementary Figure S5: Supporting data of the TLR3 specificity experiments of TL-532. (A) The endolysosomal transduction signaling pathway specificity of TL-532 was confirmed in RAMOS wt cells by adding the specific vacuolar H+-ATPase inhibitor Bafilomycin A1 (100 nM for 45 min) prior to treatment with 10 µg/mL of TLR7-ligand (CL264), 5 µg/mL of TLR9-ligand (ODN2006), 500 µg/mL of TL-532, and 0.1 µg/mL of TNFα for 24 hours. NF-κB activation was measured with SEAP reporter gene assay and expressed as the percentage of SEAP over the relative TNFα activation of the DMSO condition. **(B-C)** The endolysosomal transduction signaling pathway specificity of TL-532 was evaluated in RAW264.7 wt cells by adding the pan-cathepsin inhibitor Z-FA-FMK (20 µM for 24hr) or the specific vacuolar H^+-ATPase inhibitor Bafilomycin A1 (50 nM for 45min) prior to treatment with 100 µg/mL of TL-532 (B) or 0.1 µg/mL of LPS (C) for 24 hours. The concentration of mTNFα was measured by ELISA and expressed in fold increase of mTNFα secretion over their respective Mock conditions. Data are the mean of two (A), or three independent assays (B-C). Results are expressed as mean ± SD. Unpaired Student's *t*-test: * = $p<0.05$; ** = $p<0.01$; *** = $p<0.001$; ns: not significant. Unpaired Student's *t*-test values are compared to Mock condition unless otherwise stated.

Supplementary Figure S6

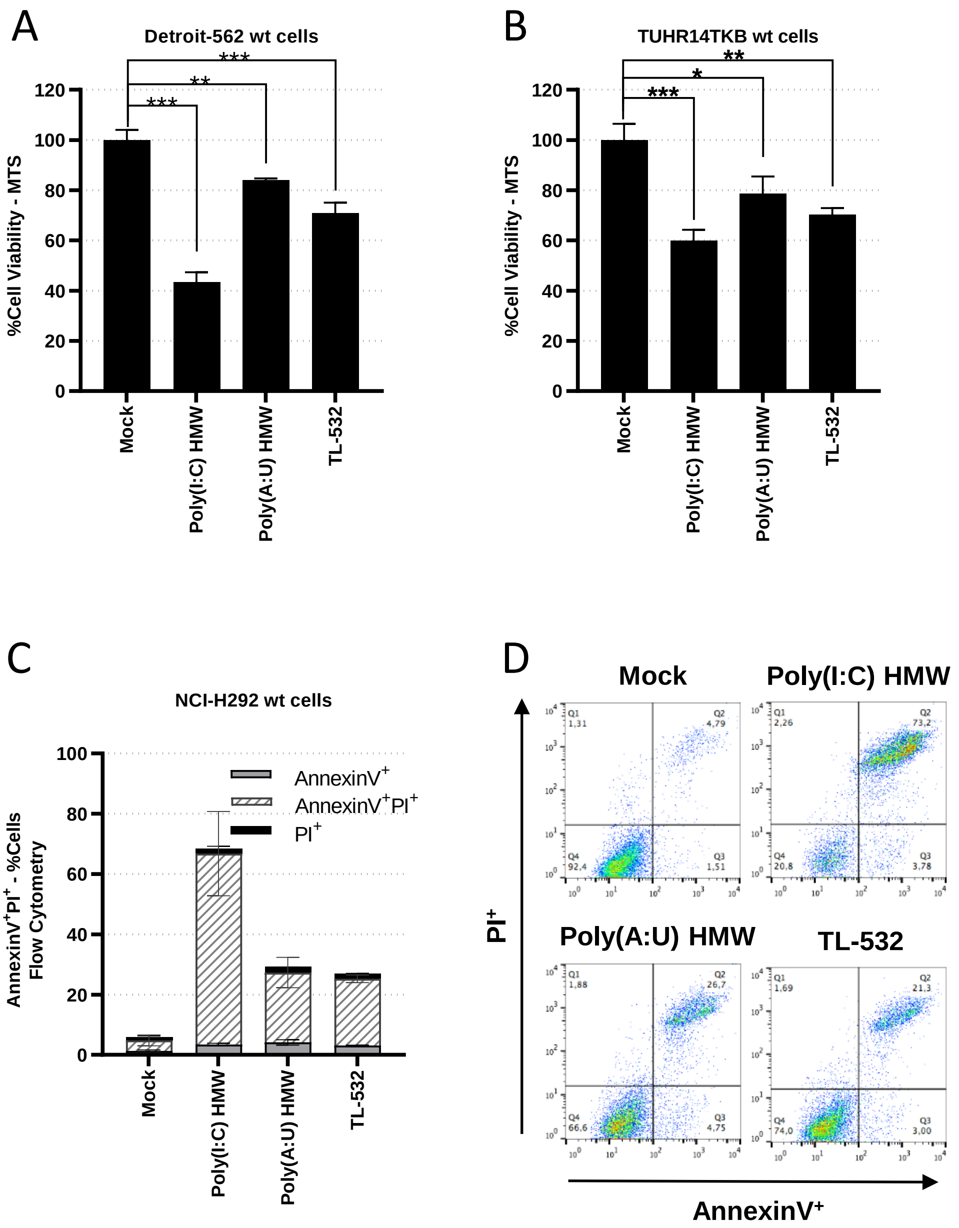

Supplementary Figure S6: TL-532 reduces cell viability and induces apoptosis in cancer cells. (A-B) Cell viability – expressed in percentage of untreated cells – was determined by MTS assay in head & neck Detroit-562 wt (A) and renal TUHR14TKB wt (B) cancer cell lines treated with Poly(I:C) HMW at 100 µg/mL, Poly(A:U) HMW and TL-532 at 500 µg/mL for 24 hours. **(C-D)** Apoptosis – expressed in % of positivity (C) or by FACS quadran stat (D) – was determined by AnnexinV^{+}PI^{+} flow cytometry staining in NCI-H292 wt cells treated with the different molecules at 5.4 µg/mL for 24 hours. Data are representative of at least two independent assays (D), or are the mean of at least two independent assays (A-C). Results are expressed as mean ± SD. Unpaired Student's *t*-test: * = p<0.05; ** = p<0.01; *** = p<0.001; ns: not significant.

Supplementary Figure S7

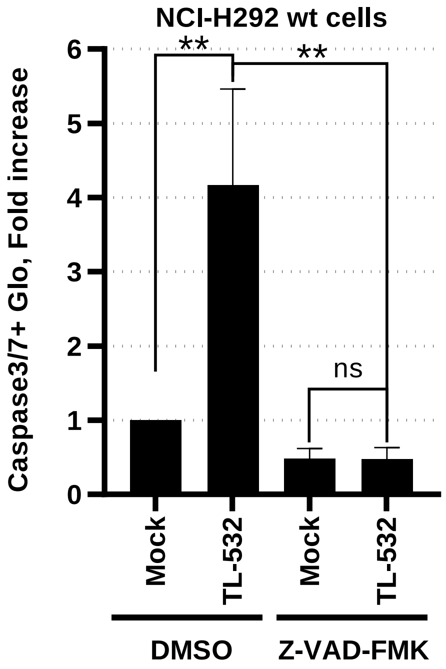

Supplementary Figure S7: TL-532 induces activation of Caspase3/7 in cancer cells. The caspase-dependent cell death was determined by adding or not the caspase inhibitor Z-VAD-FMK (20 µM for 2 hours) prior to TL-532 treatment at 100 µg/mL for 6 hours. Activation was measured with Caspase3/7-Glo assay. Data are the mean of three independent assays. Results are expressed as mean ± SD. Unpaired Student’s t-test: * = $p<0.05$; ** = $p<0.01$; *** = $p<0.001$, ns: not significant.

Supplementary Figure S8

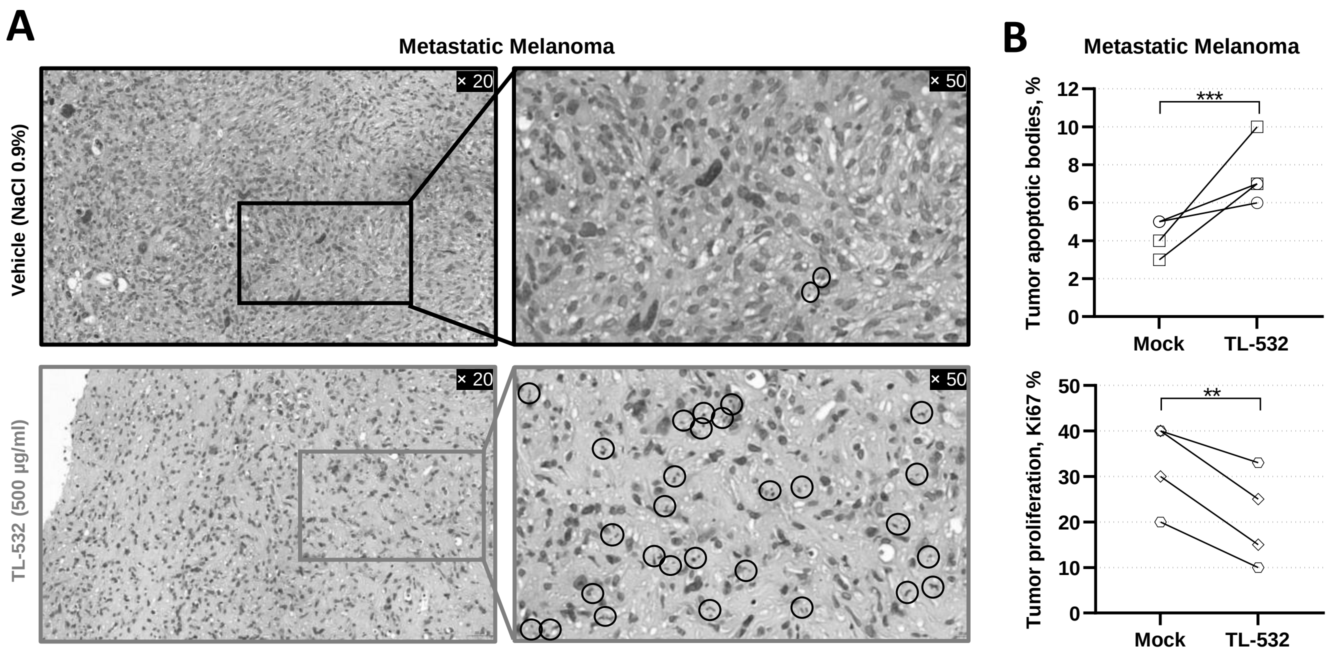

Supplementary Figure S8 : TL-532 induces cancer cell death in two *ex vivo* samples. (A-B) To analyze TL-532 activity in a more physiological model, two distinct tumors of vaginal metastasis with bladder invasion from melanoma belonging to the same patient were cultured and treated *ex-vivo* in presence or not with 500 µg/ml of TL-532 for 24 or 48 hours, before anatomopathological analysis. (A) Shows a representative field at 24h post-treatment. Apoptotic bodies are circled in black. (B) Shows the respective percentage of apoptotic bodies (upper panel) and proliferative cells (Ki67 positive cells - lower panel) over the untreated condition. Data are representative of two independent tumors from the same patient at two different time points post treatment as indicated above. Paired Student's t-test: * = $p<0.05$; ** = $p<0.01$; *** = $p<0.001$, ns: not significant.

Supplementary Figure S9

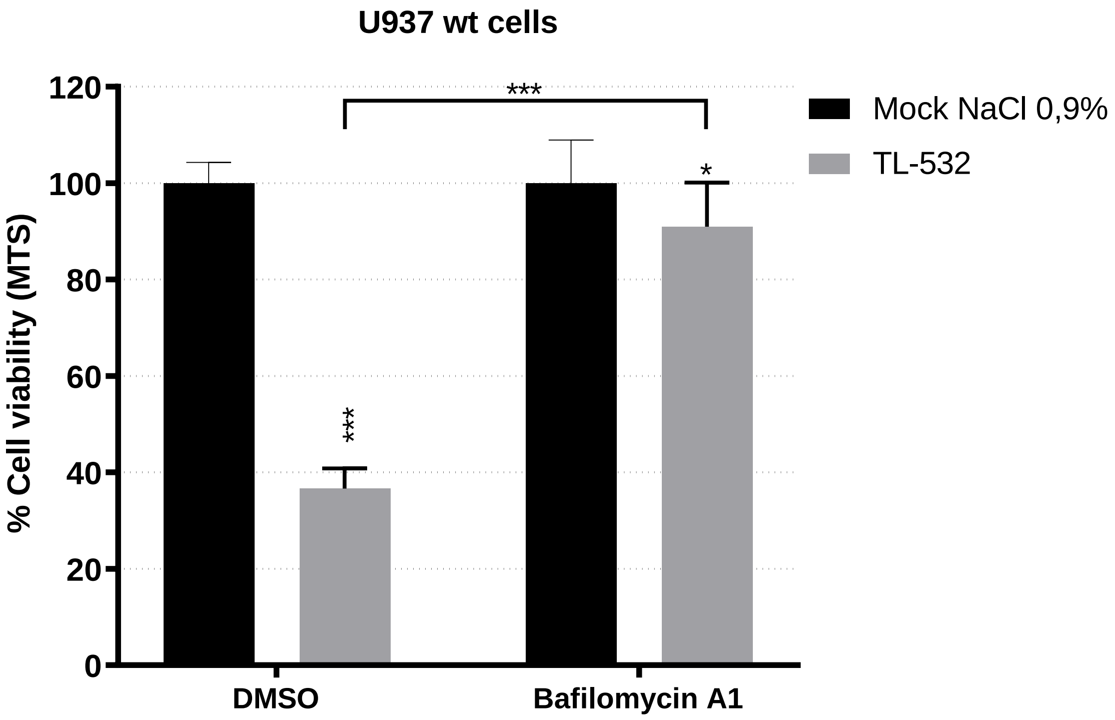

Supplementary Figure S9: TL-532 reduces cell viability in U937 wt in an endolysosomal transduction signaling pathway dependent manner. The endolysosomal transduction signaling pathway specificity of TL-532 was confirmed in U937 wt cells by adding the specific vacuolar H+-ATPase inhibitor Bafilomycin A1 (40 nM for 45 min) prior to treatment with 500 µg/mL of TL-532 for 24 hours. Cell viability was measured by MTS assay. Data are the mean of two independent assays. Results are expressed as mean ± SD. Unpaired Student's *t*-test: * = $p<0.05$; ** = $p<0.01$; *** = $p<0.001$; ns: not significant. Unpaired Student's *t*-test values are compared to Mock condition unless otherwise stated.

Supplementary Figure S10

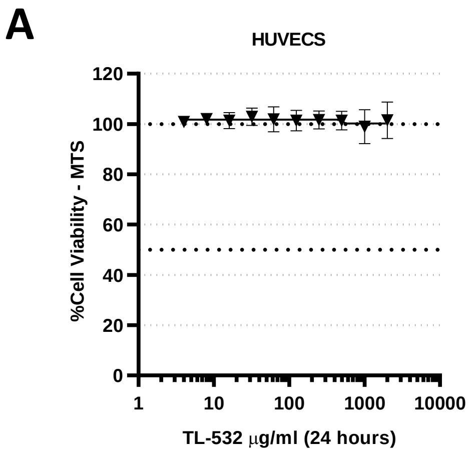

Supplementary Figure S10: TL-532 does not induce cytotoxicity in *in vitro* primary cell models. (A-B) Cell viability – expressed in percentage of untreated cells – was determined by MTS assay in Human Umbilical Vein Endothelial Cells HUVECS (A) and by LDH Release in Primary Human Hepatocytes PHH (B) treated with a dose escalation of TL-532 from 4 to 2000 µg/mL in HUVECS or from 5 to 2000 µg/mL in PHH for 24 hours. All data are the means of three independent assays. Results are expressed as mean ± 95% confidence interval.

Supplementary Table S1

dsRNA ID#	Sequence 5'→3'		% Inosine
**TL-1**	**Sense**	UACIUCAUAUAIIIACCUAUIUUAUCUICIUIUCCAACCUUAIIAUUCAC	22
	**Antisense**	IUIAAUCCUAAIIUUIIACACICAIAUAACAUAIIUCCCUAUAUIACIUA	
**TL-3**	**Sense**	UCIIUCIACICAAICIAUUACACUCCUIUCACAUCAUAAUCIUUUICUAU	23
	**Antisense**	AUAICAAACIAUUAUIAUIUIACAIIAIUIUAAUCICUUICIUCIACCIA	
**TL-4**	**Sense**	AAUIAAIUAUUIICAIACAUUIAIUICCIAACAAIACCUIACCUAACIIU	23
	**Antisense**	ACCIUUAIIUCAIIUCUUIUUCIICACUCAAUIUCUICCAAUACUUCAUU	
**TL-5**	**Sense**	CICUIUUUUCIAAAUUACCCUUUAUICICIIIUAUUIAACCACICUUAUI	23
	**Antisense**	CAUAAICIUIIUUCAAUACCCICICAUAAAIIIUAAUUUCIAAAACAICI	
**TL-6**	**Sense**	IIAAIUIUIICUAIAUCUUAICUUACIUCACUAIAIIIUCCACIUUUAIU	24
	**Antisense**	ACUAAACIUIIACCCUCUAIUIACIUAAICUAAIAUCUAICCACACUUCC	
**TL-9**	**Sense**	AAIAIAIUCUCAUAAUACIUCCIICCICAUICICAIIIUAUAUUUIIACA	25
	**Antisense**	UIUCCAAAUAUACCCUICICAUICIICCIIACIUAUUAUIAIACUCUCUU	
**TL-10**	**Sense**	AUAIAAACUACAIIACUAACCUUCCUIICAACCIIIAIIUIIIAAUCCIU	26
	**Antisense**	ACIIAUUCCCACCUCCCIIUUICCAIIAAIIUUAIUCCUIUAIUUUCUAU	
**TL-13**	**Sense**	IAIIAIIAIUCIUCAIACCAIAUAICUUUIAUIUCCUIAUCIIAAIIAUC	26
	**Antisense**	IAUCCUUCCIAUCAIIACAUCAAAICUAUCUIIUCUIACIACUCCUCCUC	
**TL-14**	**Sense**	IIAUACIAIAUCCIUAIAUUIAUAAIIIACACIIAAUAUCCCCIIACICA	26
	**Antisense**	UICIUCCIIIIAUAUUCCIUIUCCCUUAUCAAUCUACIIAUCUCIUAUCC	
**TL-16**	**Sense**	ACIUUCUAAIAIUUIIACIAAAUIUUUCICIACCUAIIAUIAIIUCICCC	26
	**Antisense**	IIICIACCUCAUCCUAIIUCICIAAACAUUUCIUCCAACUCUUAIAACIU	
**TL-17**	**Sense**	UACIUAICAAIIUIACACAAICACAIUAIAUCCUICCCICIUUUCCUAUI	26
	**Antisense**	CAUAIIAAACICIIICAIIAUCUACUIUICUUIUIUCACCUUICUACIUA	
**TL-18**	**Sense**	CUAIUUIUIIAUUIIAUUICCAUUCUCCIAIUIUAUUACCIUIACIICCI	26
	**Antisense**	CIICCIUCACIIUAAUACACUCIIAIAAUIICAAUCCAAUCCACAACUAI	
**TL-19**	**Sense**	CACIIIUCCCAUIUAAUICAIUCIUAICCUACCUIACUIUACUUIIAAIU	26
	**Antisense**	ACUUCCAAIUACAIUCAIIUAIICUACIACUICAUUACAUIIIACCCIUI	
**TL-20**	**Sense**	IACCIIACIAACCACAIAICICUIIAAIAAUCUCUAICUICUUUACAAAI	26
	**Antisense**	CUUUIUAAAICAICUAIAIAUUCUUCCAICICUCUIUIIUUCIUCCIIUC	
**TL-21**	**Sense**	UUUCCCACUICCUUAAICCIICUUICCCUUUCUICCUIUAIAUCCAUUII	26
	**Antisense**	CCAAUIIAUCUACAIICAIAAAIIICAAICCIICUUAAIICAIUIIIAAA	
**TL-22**	**Sense**	ICAACUUCIAIIACCUAAUIUIACCIACCUAIAUUCIICAUUIUIIICAI	27
	**Antisense**	CUICCCACAAUICCIAAUCUAIIUCIIUCACAUUAIIUCCUCIAAIUUIC	
**TL-23**	**Sense**	IAUCUAUIICIUIAIACCCIUUAUICUCCAUUACIIUCAIUIIIUCACAI	27
	**Antisense**	CUIUIACCCACUIACCIUAAUIIAICAUAACIIIUCUCACICCAUAIAUC	
**TL-24**	**Sense**	ACUICIACIUUCUAAACIUUIIUCCIUCAIAAICICCAUCCAIIAUCACI	28
	**Antisense**	CIUIAUCCUIIAUIICICUUCUIACIIACCAACIUUUAIAACIUCICAIU	
**TL-25**	**Sense**	ACUIIUICCAACICICAIICAUAIUUCIAIIAIAAUUAUCCIIIIICAAU	28
	**Antisense**	AUUICCCCCIIAUAAUUCUCCUCIAACUAUICCUICICIUUIICACCAIU	
**TL-26**	**Sense**	IACAACCAICAUCUCIIIUCUUICCCAACCCIUCUACACICUIUUAUAIC	28
	**Antisense**	ICUAUAACAICIUIUAIACIIIUUIIICAAIACCCIAIAUICUIIUUIUC	
**TL-27**	**Sense**	CAUICUAICIUICIIIIUACACUUICUAACCAUUUIIIACACIIIACACU	28
	**Antisense**	AIUIUCCCIUIUCCCAAAUIIUUAICAAIUIUACCCCICACICUAICAUI	
**TL-28**	**Sense**	AUAIACIIACAICUUIIUAUCCUIAICACAIUCICICIUCCIAAUCUAIC	28
	**Antisense**	ICUAIAUUCIIACICICIACUIUICUCAIIAUACCAAICUIUCCIUCUAU	
**TL-29**	**Sense**	UACCCAUACUCCACCIUUIICAIIIIIAUCICAUIUCCCACIUIAAACAU	28
	**Antisense**	AUIUUUCACIUIIIACAUICIAUCCCCCUICCAACIIUIIAIUAUIIIUA	
**TL-30**	**Sense**	AIUACAAIACUAICCUUICUAICAACCICIIICUIIIAICCUAAIIUAUC	28
	**Antisense**	IAUACCUUAIICUCCCAICCCICIIUUICUAICAAIICUAIUCUUIUACU	
**TL-31**	**Sense**	UUCAICICICAIICUUIIIUCIAIAUAAAAUCUCCAIUICCCAAIACCAC	28
	**Antisense**	IUIIUCUUIIICACUIIAIAUUUUAUCUCIACCCAAICCUICICICUIAA	
**TL-32**	**Sense**	ICAACIIAACIUCCUUAICUCCIICAIICAAUUAAIIIIAACICAAICAU	28
	**Antisense**	AUICUUICIUUCCCCUUAAUUICCUICCIIAICUAAIIACIUUCCIUUIC	
**TL-33**	**Sense**	IUAUCAUUIUICACCUICCIIUIACCACUCAACIAUIUIIIIACICCIUU	29
	**Antisense**	AACIICIUCCCCACAUCIUUIAIUIIUCACCIICAIIUICACAAUIAUAC	

Supplementary Table S1

TL-34	Sense	IUUACCCAUAUIIUCCACAIIACACUCIUCICUUCCIIICUUICCCUCUA	29
	Antisense	UAIAIIICAAICCCIIAAICIACIAIUIUCCUIUIIACCAUAUIIIUAAC	
TL-36	Sense	ACICUIUCUCUIICACIUIIIUIICCUAIAIIAAUCACAUCCAAICCUII	30
	Antisense	CCAIICUUIIAUIUIAUUCCUCUAIICCACCCACIUICCAIAIACAICIU	
TL-37	Sense	IUCIUIICAAUIUUCIUCUIIIUIUIIUCUACACAAUICIIICIIUICIU	30
	Antisense	ACICACCICCCICAUUIUIUAIACCACACCCAIACIAACAUUICCACIAC	
TL-38	Sense	UIICAIACACACCIUIACCCCICCUCUCCAUUIAUICCACIICIAAUIUC	31
	Antisense	IACAUUCICCIUIICAUCAAUIIAIAIICIIIIUCACIIUIUIUCUICCA	
TL-40	Sense	AICCCUUCUCCCCUICIICCACICCCIUAIAIAUCACICCUUUIACCCUC	33
	Antisense	IAIIIUCAAAIICIUIAUCUCUACIIICIUIICCICAIIIIAIAAIIICU	
TL-41	Sense	ACICUICAIIACUUICAACCIIICAIACUCIICIICAIIUCCUAIUICAI	33
	Antisense	CUICACUAIIACCUICCICCIAIUCUICCCIIUUICAAIUCCUICAICIU	
TL-42	Sense	IICIAAIICCCUAACIIIAIAUACICICCCACAACUCIICICIAAUACII	33
	Antisense	CCIUAUUCICICCIAIUUIUIIICICIUAUCUCCCIUUAIIICCUUCICC	
TL-44	Sense	ICACCAIAUCUIUAAIIUCCICCACICAIACIAIICCIIICIIAIACCAC	34
	Antisense	IUIIUCUCCICCCIICCUCIUCUICIUIICIIACCUUACAIAUCUIIUIC	
TL-45	Sense	UCCUIIAIIAIIIICIIAUAICCUCUUACCCIUICCCCACCIUUIICIIU	34
	Antisense	ACCICCAACIIUIIIICACIIIUAAIAIICUAUCCICCCCUCCUCCAIIA	
TL-47	Sense	UICICCIIUCCCCAICCICICUCAUICUCIICACCICCAUAACCAIACCI	36
	Antisense	CIIUCUIIUUAUIICIIUICCIAICAUIAICICIICUIIIIACCIICICA	
TL-48	Sense	UAICCICCCCUIIICCICIIUCCICUACCUUICAIIAAUCIAIICCIUCC	36
	Antisense	IIACIICCUCIAUUCCUICAAIIUAICIIACCICIICCCAIIIICIICUA	
TL-49	Sense	ICUICUUCIICICCCCIIICICACCCCUICCICIIIIICIIIAUCICCCI	43
	Antisense	CIIICIAUCCCICCCCCICIICAIIIIUICICCCIIIICICCIAAICAIC	
TL-50	Sense	IIAUAIIIICIICCCCIIICIICIICCICCIICCCCIICIICIIIICIUU	46
	Antisense	AACICCCCICCICCIIIICCIICIICCICCICCCIIIICCICCCCUAUCC	
TL-51	Sense	IUCIICICCCIICCCCCCIICCCCICAICIIICUCCCCICCCIIICCICC	47
	Antisense	IICIICCCIIICIIIIAICCCICUICIIIICCIIIIIICCIIICICCIAC	
TL-52	Sense	IIIIIICCCACICIICIICICCICCIICICCCCCIIIICICCCCICIUCI	48
	Antisense	CIACICIIIICICCCCIIIIICICCIICIICICCICCICIUIIICCCCCC	
TL-53	Sense	CIIICCIICIIIIIICIIICIIIIICCCCUIICCCICCCIICICCCCICI	49
	Antisense	CICIIIICICCIIICIIICCAIIIICCCCCICCCICCCCCCICCIICCCI	
TL-54	Sense	IIICCIIIICIICCCCIIICIICIICCICCIICCCCIICIICIIIICICC	50
	Antisense	IICICCCCICCICCIIIICCIICIICCICCICCCIIIICCICCCCIICCC	
TL-411	Sense	IIIIIIIIIIIIIIIIIIIIIIIIIIIIIIIIIIIIIIIIIIIIIIIIII	50
	Antisense	CCCCCCCCCCCCCCCCCCCCCCCCCCCCCCCCCCCCCCCCCCCCCCCCCC	
TL-412	Sense	AAAAAAAAAAAAAAAAAAAAAAAAAAAAAAAAAAAAAAAAAAAAAAAAAA	0
	Antisense	UUUUUUUUUUUUUUUUUUUUUUUUUUUUUUUUUUUUUUUUUUUUUUUUUU	
TL-413*	Sense	AUUUAAAAAAAUAAAUAUAAUAAAAUAUAAUUUAAUUAAUUAUUUAUUAAU	0
	Antisense	AUUAAUAAAUAAUUAAUUAAAUUAUAUUUUAUUAUAUUUAUUUUUUAAAU	

Supplementary Table S1: Sequence list of the 47 tested 50bp dsRNAs. dsRNAs TL-1 to -48 were manufactured by IDT. dsRNAs TL-49 to -54, and -411 to -413 were manufactured by Horizon Discovery. All dsRNAs have a phosphate backbone, bear 5’-OH and 3’-OH ends at the two strands, and do not have other modifications. All of the tested dsRNAs were chemically manufactured on solid-phase support and purified by chromatography. *: 50% adenine and 50% uracil content, the same %(A:U) as TL-412.

Supplementary Table S2

dsRNA ID#<br>*Common name*	Sequence 5'→3'	
**TL-432**<br>*A70*	**Sense 70 bases**	AAAAAAAAAAAAAAAAAAAAAAAAAAAAAAAAAAAAAAAA<br>AAAAAAAAAAAAAAAAAAAAAAAAAAAAAA
	**Antisense 70 bases**	UUUUUUUUUUUUUUUUUUUUUUUUUUUUUUUUUUUUUUUU<br>UUUUUUUUUUUUUUUUUUUUUUUUUUUUUU
**TL-452**<br>*A90*	**Sense 90 bases**	AAAAAAAAAAAAAAAAAAAAAAAAAAAAAAAAAAAAAAAA<br>AAAAAAAAAAAAAAAAAAAAAAAAAAAAAAAAAAAAAAAA<br>AAAAAAAAAA
	**Antisense 90 bases**	UUUUUUUUUUUUUUUUUUUUUUUUUUUUUUUUUUUUUUUU<br>UUUUUUUUUUUUUUUUUUUUUUUUUUUUUUUUUUUUUUUU<br>UUUUUUUUUU
**TL-532**<br>*I10 A50 I10*	**Sense 70 bases**	IIIIIIIIIIAAAAAAAAAAAAAAAAAAAAAAAAAAAAAA<br>AAAAAAAAAAAAAAAAAAAAIIIIIIIIII
	**Antisense 70 bases**	CCCCCCCCCCUUUUUUUUUUUUUUUUUUUUUUUUUUUUUU<br>UUUUUUUUUUUUUUUUUUUUCCCCCCCCCC
**TL-533**<br>*A35 I35*	**Sense 70 bases**	AAAAAAAAAAAAAAAAAAAAAAAAAAAAAAAAAAAIIIII<br>IIIIIIIIIIIIIIIIIIIIIIIIIIIIII
	**Antisense 70 bases**	CCCCCCCCCCCCCCCCCCCCCCCCCCCCCCCCCCCUUUUU<br>UUUUUUUUUUUUUUUUUUUUUUUUUUUUUU
**TL-534**<br>*A10 I50 A10*	**Sense 70 bases**	AAAAAAAAAAIIIIIIIIIIIIIIIIIIIIIIIIIIIIII<br>IIIIIIIIIIIIIIIIIIIIAAAAAAAAAA
	**Antisense 70 bases**	UUUUUUUUUUCCCCCCCCCCCCCCCCCCCCCCCCCCCCCC<br>CCCCCCCCCCCCCCCCCCCCUUUUUUUUUU
**TL-535**<br>*I70*	**Sense 70 bases**	IIIIIIIIIIIIIIIIIIIIIIIIIIIIIIIIIIIIIIII<br>IIIIIIIIIIIIIIIIIIIIIIIIIIIIII
	**Antisense 70 bases**	CCCCCCCCCCCCCCCCCCCCCCCCCCCCCCCCCCCCCCCC<br>CCCCCCCCCCCCCCCCCCCCCCCCCCCCCC
**TL-536**<br>*random 70pb*	**Sense 70 bases**	UACIUCAUAUAIIIACCUAUIUUAUCUICIUIUCCAACCU<br>UAIIAUUCACUCIIUCIACICAAICIAUUA
	**Antisense 70 bases**	UAAUCICUUICIUCIACCIAIUIAAUCCUAAIIUUIIACA<br>CICAIAUAACAUAIIUCCCUAUAUIACIUA
**TL-537**<br>*I5 A60 I5*	**Sense 70 bases**	IIIIIAAAAAAAAAAAAAAAAAAAAAAAAAAAAAAAAAAA<br>AAAAAAAAAAAAAAAAAAAAAAAAAIIIII
	**Antisense 70 bases**	CCCCCUUUUUUUUUUUUUUUUUUUUUUUUUUUUUUUUUUU<br>UUUUUUUUUUUUUUUUUUUUUUUUUCCCCC
**TL-538**<br>*I15 A40 I15*	**Sense 70 bases**	IIIIIIIIIIIIIIIAAAAAAAAAAAAAAAAAAAAAAAAA<br>AAAAAAAAAAAAAAAIIIIIIIIIIIIIII
	**Antisense 70 bases**	CCCCCCCCCCCCCCCUUUUUUUUUUUUUUUUUUUUUUUUU<br>UUUUUUUUUUUUUUUCCCCCCCCCCCCCCC
**TL-539**<br>*I20 A30 I20*	**Sense 70 bases**	IIIIIIIIIIIIIIIIIIIIAAAAAAAAAAAAAAAAAAAA<br>AAAAAAAAAAIIIIIIIIIIIIIIIIIIII
	**Antisense 70 bases**	CCCCCCCCCCCCCCCCCCCCUUUUUUUUUUUUUUUUUUUU<br>UUUUUUUUUUCCCCCCCCCCCCCCCCCCCC
**TL-540**<br>*I25 A20 I25*	**Sense 70 bases**	IIIIIIIIIIIIIIIIIIIIIIIIIAAAAAAAAAAAAAAA<br>AAAAAIIIIIIIIIIIIIIIIIIIIIIIII
	**Antisense 70 bases**	CCCCCCCCCCCCCCCCCCCCCCCCCUUUUUUUUUUUUUUU<br>UUUUUCCCCCCCCCCCCCCCCCCCCCCCCC
**TL-541**<br>*I30 A10 I30*	**Sense 70 bases**	IIIIIIIIIIIIIIIIIIIIIIIIIIIIIIAAAAAAAAAA<br>IIIIIIIIIIIIIIIIIIIIIIIIIIIIII
	**Antisense 70 bases**	CCCCCCCCCCCCCCCCCCCCCCCCCCCCCCUUUUUUUUUU<br>CCCCCCCCCCCCCCCCCCCCCCCCCCCCCC

Supplementary Table S2: Sequence list of the tested 70 and 90bp dsRNAs. TL-432 and -452 were Poly(A:U) dsRNA of 70 and 90bp, respectively. The sequences of the 70bp dsRNAs TL-532 thru -535 contained an increasing amount of Poly(I:C) compared to TL-432. TL-536 was a random 70bp sequence. The sequences of TL-537 thru -541 contained different amount of Poly(I:C) compared to TL-532. All dsRNAs have a phosphate backbone, bear 5'-OH and 3'-OH ends at the two strands, and do not have other modifications. All listed dsRNAs were manufactured by Horizon Discovery and NittoAVECIA and were chemically manufactured on solid-phase support and purified by chromatography.
